# Comorbidities predict institutionalization and mortality in biomarker-confirmed alzheimer’s disease

**DOI:** 10.1186/s13195-025-01807-6

**Published:** 2025-07-12

**Authors:** Xin Xia, Alice Clark, Niels Juul Brogaard, Alex Mourer, Anna Areovimata, Maria Eriksdotter, Henrik Zetterberg, Silke Kern, Tobias Skillbäck, Linus Jönsson

**Affiliations:** 1 Department of Neurobiology, Care Sciences and Society, Section for Neurogeriatrics, Karolinska Institutet, Solna, Sweden; 2 Novo Nordisk A/S, Bagsvaerd, Denmark; 3 Division of Clinical Geriatrics, Department of Neurobiology, Care Sciences and Society, Karolinska Institutet, Huddinge, Sweden; 4 Theme Inflammation and Aging, Karolinska University Hospital, Huddinge, Sweden; 5 Department of Psychiatry and Neurochemistry, Institute of Neuroscience and Physiology, Sahlgrenska Academy, University of Gothenburg, Göteborg, Sweden; 6 Clinical Neurochemistry Laboratory, Sahlgrenska University Hospital, Göteborg, Sweden; 7 Department of Neurodegenerative Disease, UCL Institute of Neurology, London, UK; 8 UK Dementia Research Institute at UCL, London, UK; 9 Hong Kong Center for Neurodegenerative Diseases, Hong Kong, China; 10 Wisconsin Alzheimer’s Disease Research Center, School of Medicine and Public Health, University of Wisconsin, University of Wisconsin-Madison, Madison, USA; 11 Neuropsychiatric Epidemiology Unit, Department of Psychiatry and neurochemistry, Sahlgrenska Academy, University of Gothenburg, Göteborg, Sweden; 12 Region Västra Götaland, Department of Neuropsychiatry, Sahlgrenska University Hospital, Göteborg, Sweden; 13 Clinical Dementia Research, Department of Psychiatry and neurochemistry, Sahlgrenska Academy, University of Gothenburg, Göteborg, Sweden; 14 Karolinska Institutet, Solnavägen 30, floor 10, BioClinicum, Solna, 171 64 Sweden

**Keywords:** Alzheimer’s disease, Biomarker, Comorbidity, Prognosis, Risk prediction, Cohort study, Register-based study

## Abstract

**Background:**

We explored the associations of comorbidities with cognitive deterioration, institutionalization, and mortality in biomarker-confirmed Alzheimer’s disease (AD) dementia.

**Methods:**

We conducted a Swedish Register-based cohort study consisting of 10,857 people (mean age 74 years) with diagnosed dementia and positive AD biomarkers (CSF Aβ_42_/P-tau_181_ ratio). Cognitive function was measured by mini-mental state examination (MMSE). Comorbidities by human body organ systems (e.g., diseases of the circulatory system) and six selected comorbidities: type-2 diabetes (T2DM), ischemic heart disease (IHD), stroke, chronic kidney disease (CKD), inflammatory bowel disease, and depression, were analyzed. Multistate Cox regressions assessed the associations of comorbidities with cognitive deterioration, institutionalization, and death.

**Results:**

Only T2DM and IHD were associated with cognitive deterioration. Mental disorders, T2DM, and stroke were linked to higher hazards of institutionalization. Endocrine-metabolic disorders, circulatory system diseases, and CKD were associated with higher mortality rates.

**Conclusions:**

Comorbidities may help inform the prognosis of biomarker-confirmed AD dementia.

**Supplementary Information:**

The online version contains supplementary material available at 10.1186/s13195-025-01807-6.

## Background

Alzheimer’s disease (AD) is a neurodegenerative disease characterized by extracellular amyloid-β (Aβ) plaques and intracellular neurofibrillary tangles in the brain [1]. AD accounts for 60-80% of dementia and is a leading cause of disability and mortality in older adults [1, 2].

Until recently, pharmacological treatments for dementia primarily focused on symptom management, but disease-modifying therapies for AD that target Aβ have emerged and been approved in recent years [3]. However, these disease-modifying therapies may not be suitable for AD patients who have high comorbidity burdens [4]. Clinical trials for the new disease-modifying therapies excluded patients with other pathologies that contribute to cognitive decline [5, 6]. AD primarily affects older adults, in whom multiple medical conditions often coexist, which leads to a reduced number of AD patients who can benefit from the new interventions [7, 8]. Understanding the role of comorbidities in AD progression is important for developing future interventions that target these comorbidities to improve AD prognosis. Investigating the role of comorbidities in AD progression is also important for the development of prediction models for AD, which can be then used in economic evaluations of novel AD treatments as well as clinical decision support systems that can inform the likely prognosis of people with AD [9, 10].

Previous studies on the associations between comorbidities and AD prognosis have mainly focused on cognitive decline and mortality [11–23]. The findings in studies on comorbidities and cognitive decline in AD have been inconsistent, with some research suggesting that comorbidities may accelerate or decelerate cognitive decline, while others indicate no significant association [11–15]. The associations between comorbidities and mortality in people with AD were more consistent in previous studies [16–18, 20–23]. In addition to cognitive decline and mortality, institutionalization is also a relevant health outcome on the disease trajectory of AD. Institutionalization reflects functional decline and loss of dependence and is a significant component of the healthcare costs of AD [24]. However, few studies have investigated the associations between comorbidities and future institutionalization in AD, yielding variable findings [20, 25, 26]. It is noteworthy that previous studies on comorbidities and AD prognosis often included a limited number of disease or summary measures, such as the Charlson comorbidity index (CCI), which focuses on predicting mortality and lacks a comprehensive view of comorbidities in AD [18, 23, 25]. In addition, data on the impact of comorbidities on biomarker-confirmed AD, which is especially relevant in the context of anti-amyloid disease-modifying treatments and important for understanding the progression of AD, is lacking.

Therefore, we conducted a cohort study using Swedish registers that include comprehensive assessments of prognostic factors and repeated information on cognition to explore the associations between a broad range of comorbidities and important events in the disease trajectory of biomarker-confirmed AD, including cognitive deterioration, institutionalization, and mortality.

## Methods

### Study design and study population

We conducted a register-based cohort study with an observational period between January 1st, 2007, and December 31st, 2020. The study received approval from the Swedish Ethical Review Authority (DNR: 2023-06581-02) and was performed in accordance with the Declaration of Helsinki.

The study population was derived from the Swedish Register of Cognitive/Dementia Disorders (SveDem). SveDem covers almost all specialist memory clinics as well as many primary care centers in Sweden and continuously registers data on patients with dementia and, since 2021, data on patients with mild cognitive impairment (MCI) [27]. By 2012, SveDem had achieved a coverage rate of 93% of specialist memory clinics and 60% of primary care centers in Sweden, with coverage continuing to increase in subsequent years [27].

SveDem includes information on the clinical diagnosis, such as AD dementia or Dementia with Lewy bodies, and cognitive tests, mainly the Mini-Mental State Examination (MMSE) [27]. The Swedish guidelines recommend the following evaluations in the basic dementia work-up: structured interviews with caregivers or relatives, MMSE and clock-drawing test, or Rowland Universal Dementia Assessment Scale, a structured evaluation of functional abilities, and computed tomography (performed in 87% of cases) or magnetic resonance imaging (performed in 16% of cases) [28, 29]. In extended dementia investigations, neuropsychological tests covering multiple cognitive domains, cerebrospinal fluid (CSF, used in 40% of cases in specialist care) biomarkers, and other neuroimaging exams such as fluorodeoxyglucose-positron emission tomography are performed in specialist memory centers [28].

A subset of the individuals in SveDem underwent CSF biomarker measurements, and CSF data of 14.5% of the individuals were available in the Sahlgrenska University Clinical Chemistry database and were used to define biomarker-confirmed AD [30, 31]. All CSF analyses were performed by certified laboratory technicians following a standard procedure, as previously reported [30, 31]. In this study, Aβ_42_/P-tau_181_ ratio was used to define biomarker-confirmed AD, as Aβ_42_/Aβ_40_ (another commonly used measure) was largely missing.

Since there is no established cut-off for Aβ_42_/P-tau_181_ ratio, we determined an optimal cut-off by maximizing the Youden index for predicting amyloid positivity, defined as an Aβ_42_/Aβ_40_ ratio below 0.072. This optimization was performed using the R package “cutpointr”, resulting in a cut-off value of 14.887 for the Aβ_42_/P-tau_181_ ratio [32].

**Fig. 1:**
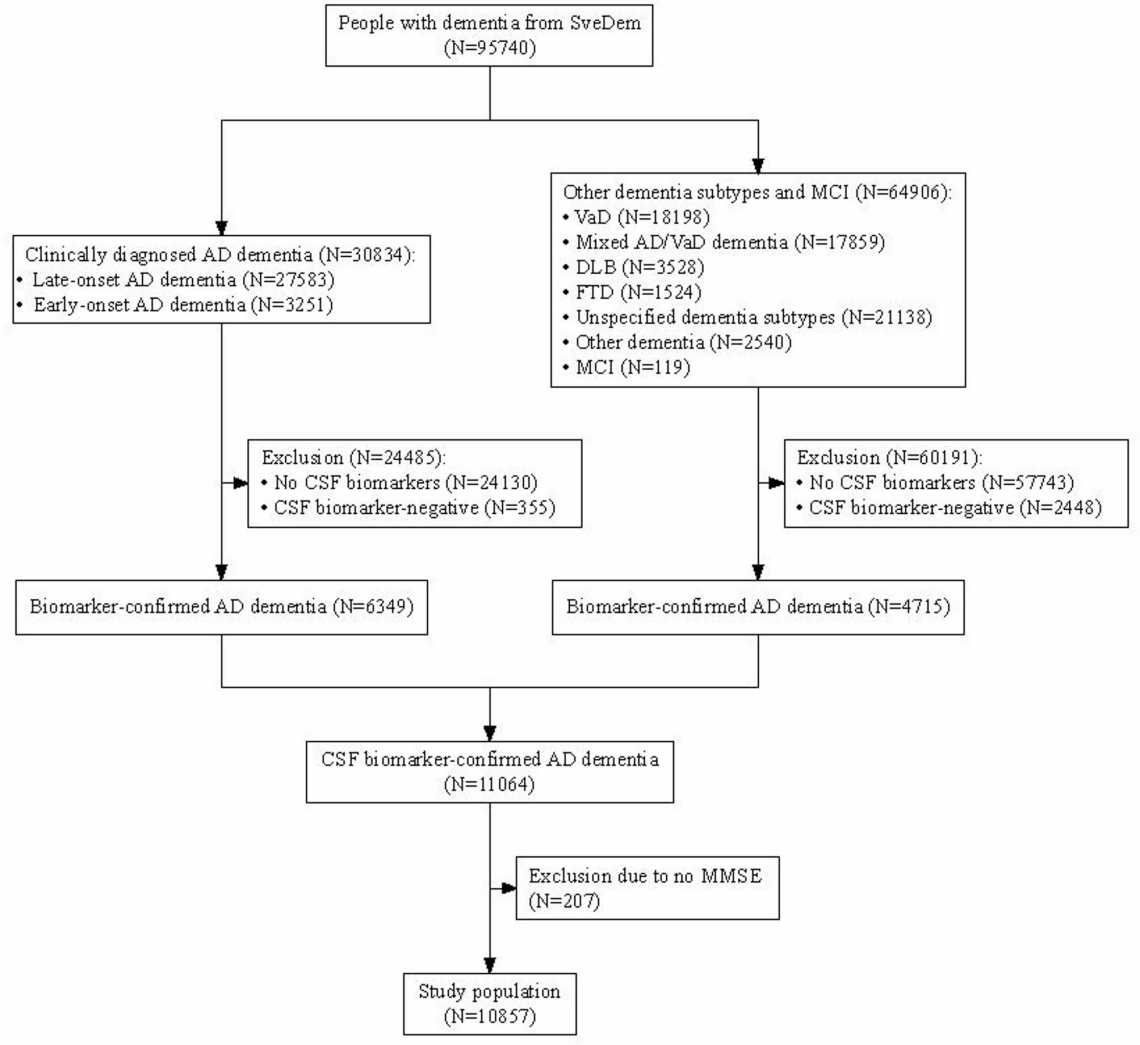
Flowchart of study participants Note: CSF Aβ status was determined by the Aβ_42_/P-tau_181_ ratio with a cut-off value of 14.887

This study included 6349 individuals with a clinical diagnosis of AD dementia who were biomarker-positive, along with 4715 biomarker-positive individuals diagnosed with other dementia subtypes or MCI (Fig. 1). After further excluding 207 people without any MMSE measurements, the final study population comprised 10,857 participants with CSF biomarker-confirmed AD dementia.

### Ascertainment of comorbidities

Comorbidities were identified by the Swedish National Patient Register (NPR), which reached national coverage of diagnoses in inpatient and specialist outpatient care settings in 2001 but not diagnoses from primary care [33]. The NPR also covers psychiatric care and emergency care from 2010 to 2016, respectively [33]. The primary diagnoses and secondary diagnoses were recorded using the 10th revision of the International Classification of Diseases (ICD-10) codes of each healthcare visit, and the dates of the visits were recorded in the NPR. In this study, comorbidities were categorized into groups based on ICD-10 chapters, primarily organized by human organ systems.

In addition, we specifically evaluated the associations of AD prognosis with the following six comorbidities because of their close links to AD: chronic kidney disease (CKD, ICD-10 code: N18), depression (ICD-10 codes: F32, F33), inflammatory bowel disease (IBD, ICD-10 codes: K50, K51), ischemic heart disease (IHD, ICD-10 codes: I20, I21, I22, I23, I24, and I25), type 2 diabetes (T2DM, ICD-10 code: E11), and stroke (ICD-10 codes: I60, I61, I62, I63, and I64). IHD, T2DM, and stroke were of special interest because they are well-established risk factors for the development of AD and cognitive decline [34]. CKD and IBD have been increasingly researched due to their possible detrimental effects on brain health and cognition [35, 36]. Depression has important implications for AD, as it is a risk factor for the development of dementia, an early sign of dementia, and a common neuropsychiatric symptom in people living with dementia [37, 38]. The sensitivities of NPR capturing the six comorbidities have been reported previously, with good sensitivities for IHD (around 80%) and stroke (> 80%) but poor sensitivity for T2DM (< 30%) [39]. The positive predictive values of diagnoses in NPR are high for IHD, stroke, and IBD (> 90%) but lower for T2DM (> 70%) [39, 40]. The validity of NPR in identifying depression and CKD is unknown.

ICD-10 codes related to MCI and dementia (F00, F01, F02, F03, F05, F06, G30, G31, and R41) were excluded when identifying comorbidities in NPR.

### Ascertainment of cognitive deterioration, institutionalization, and mortality

Cognitive deterioration was defined as progression from a less severe to a more severe MMSE-defined stage of AD dementia. MMSE data was collected in SveDem. According to the National Board of Health and Welfare’s dementia care guidelines, annual follow-up examinations, including cognitive assessments, are recommended for individuals diagnosed with dementia [28]. Consequently, patients undergoing follow-up evaluations in SveDem would have longitudinal MMSE data. The stage of AD dementia was classified using MMSE scores as follows: very mild (MMSE = 26-≤30), mild (MMSE = 21–25), moderate (MMSE = 11–20), and severe (MMSE < 11).

Institutionalization status was ascertained from the National Register of Care and Social Services for the Elderly and Persons with Impairments, which includes monthly reported information on whether a person is living at ordinary residence or is institutionalized [41].

Vital status and the date of death were ascertained through the Swedish Cause of Death Register [42].

### Statistical analysis

Descriptive statistics (e.g., means for continuous variables, proportions for categorical variables) were calculated for demographic and other characteristics by MMSE-defined AD dementia stages at the time of MCI or dementia diagnosis (index date).

**Fig. 2:**
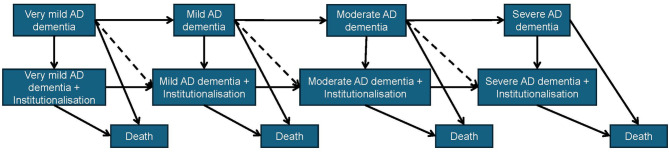
Possible states and transitions in the multistate models Note: The transitions indicated by the dashed lines were excluded due to too few cases (less than three)

We applied multistate Cox proportional hazard models to analyze the associations of comorbidities with transitions across different states of AD dementia stages, institutionalization, and death (Fig. 2). The multistate Cox proportional hazard models in this study can be seen as a set of Cox proportional hazard models stratified by the transitions.

The timescale for the multistate Cox proportional hazard models was the time since entering SveDem (i.e., follow-up time from the index date). Each individual was followed until (1) death, (2) the end of the observational period (December 31st, 2020), or (3) without MMSE assessments for 15 consecutive months following the last recorded MMSE assessment, except in cases of severe AD dementia, whichever came first. All Cox proportional hazard models were adjusted for age and sex.

To identify ICD-10 chapter-based comorbidity groups that could predict state transitions, we followed these analytical steps: (1) each comorbidity chapter was individually included in separate multistate models, (2) all comorbidity chapters associated with the prognosis of AD dementia in the first step were included together in a single multistate model, and (3) the statistically significant comorbidity chapters from the second step were incorporated into the final multistate models. For the six selected comorbidities, we included all of them simultaneously in the multistate models to evaluate their associations with possible transitions shown in Fig. 2.

Given that our preliminary analyses indicated that the occurrences of comorbidities may differ across the various stages of AD dementia (Supporting information Figure S1 and Figure S2), we incorporated all comorbidities as time-varying covariates in the multistate models to capture the evolving comorbidity profiles at different stages of AD dementia.

We conducted sensitivity analyses by further adjusting the multistate models for log-transformed CSF Aβ_42_, P-tau_181_, and T-tau levels at baseline (i.e., the time of AD diagnosis), as these biomarkers can predict the rate of cognitive decline in individuals with AD dementia, and their levels may differ across various comorbidity profiles [43]. The levels of these CSF biomarkers at the time of AD diagnosis may differ across different comorbidity profiles because they can contribute to cognitive impairment through various mechanisms other than AD pathologies, which may lead to similar levels of cognitive impairment given lower AD pathology burden [34, 44].

R version 4.3.3 and packages “survival” and “mstate” were used for the analyses [45, 46]. As the study is an exploratory study aiming to identify comorbidities that can inform or potentially influence the prognosis in AD and to reduce the risk of missing comorbidities that might be important predictors for prognosis in AD or possible targets for improving AD prognosis, we did not make specific corrections for multiple comparisons [47, 48].

## Results

### Characteristics of the study population

The study population mainly consisted of people with very mild to moderate dementia, and the proportion of females was higher than males (Table 1). Most people received a diagnosis in specialist care, and AD dementia was the most common diagnosis (Table 1). Few people were institutionalized at index dates (Table 1).

**Table 1:** Characteristics of people with biomarker-confirmed AD dementia by the stages of AD dementia at index dates

	Very mild AD dementia based on Aβ_42_/*P*-tau_181_ ratio (*N* = 2809)	Mild AD dementia based on Aβ_42_/*P*-tau_181_ ratio (*N* = 4507)	Moderate AD dementia based on Aβ_42_/*P*-tau_181_ ratio (*N* = 3084)	Severe AD dementia based on Aβ_42_/*P*-tau_181_ ratio (*N* = 349)
**MMSE**,** mean (SD)**	27.33 (1.20)	23.08 (1.40)	17.17 (2.50)	5.08 (4.04)
**Age (years)**,** mean (SD)**	73.46 (7.41)	74.07 (7.65)	74.07 (8.02)	73.57 (8.17)
**Sex-female**,** n (%)**	1484 (52.83%)	2519 (55.89%)	1797 (58.27%)	195 (55.87%)
**Diagnostic setting**,** n (%)**				
Primary care	384 (13.67%)	669 (14.84%)	455 (14.75%)	76 (21.78%)
Specialist care	2425 (86.33%)	3838 (85.16%)	2629 (85.25%)	273 (78.22%)
**Diagnosis registered in SveDem**,** n (%)**				
Late-onset AD dementia	1324 (47.13%)	2006 (44.51%)	1231 (39.92%)	127 (36.39%)
Early-onset AD dementia	410 (14.60%)	572 (12.69%)	438 (14.20%)	58 (16.62%)
Mixed AD dementia with VaD	403 (14.35%)	740 (16.42%)	559 (18.13%)	56 (16.05%)
FTD	75 (2.67%)	72 (1.60%)	50 (1.62%)	15 (4.30%)
VaD	190 (6.76%)	353 (7.83%)	244 (7.91%)	26 (7.45%)
LBD	68 (2.42%)	152 (3.37%)	95 (3.08%)	9 (2.58%)
PDD	20 (0.71%)	32 (0.71%)	20 (0.65%)	5 (1.43%)
Other dementia	56 (1.99%)	77 (1.71%)	57 (1.85%)	10 (2.87%)
Unspecified dementia	239 (8.51%)	492 (10.92%)	390 (12.65%)	43 (12.32%)
MCI	24 (0.85%)	11 (0.24%)	0 (0.00%)	0 (0.00%)
**Living arrangement**,** n (%)**				
Institutionalized	24 (0.85%)	51 (1.13%)	102 (3.31%)	43 (12.32%)
Non-institutionalized	2785 (99.15%)	4456 (98.87%)	2982 (96.69%)	306 (87.68%)
**Aβ₄₂/P-tau₁₈₁ ratio**,** mean (SD)**	6.66 (3.13)	6.65 (3.16)	6.47 (3.15)	6.36 (3.16)

Abbreviations: AD = Alzheimer’s disease; FTD = Frontotemporal dementia; LBD = Lewy body dementia; MCI = mild cognitive impairment; MMSE = mini-mental state examination; PDD = Parkinson’s disease dementia; SD = standard deviation; VaD = vascular dementia

Note: 108 people did not have MMSE at index dates

The characteristics of individuals from SveDem who were included and excluded from this study were described in Table S1. Compared with people who were included in the study, people who were excluded due to missing data on CSF Aβ status were older, had higher prevalences of T2DM, IHD, CKD, and stroke but a lower prevalence of depression, and were more often prescribed with anticoagulant or antiplatelet medications within 3 months of index dates (Table S1). Individuals who were excluded due to negative CSF Aβ were younger, had higher prevalences of T2DM, stroke, and depression, and were more often prescribed anticoagulant or antiplatelet medications than individuals included in the study (Table S1). Those who were excluded due to missing MMSE had higher prevalences of IHD and stroke and were more often prescribed anticoagulant or antiplatelet medications than people included in the study (Table S1).

In biomarker-confirmed AD dementia, the most common comorbidities were diseases of the circulatory system (affecting approximately 40%), followed by diseases of the eye and adnexa (over 30%), and diseases of the musculoskeletal system and connective tissue (around 30%) (Table S2). T2DM, depression, and IHD were also common (each present in > 5% of individuals), whereas stroke (< 5%), CKD (< 2%), and IBD (approximately 1%) were relatively rare in people with biomarker-confirmed AD dementia (Table S2). Notably, the validity of NPR in identifying depression and CKD may be limited.

### The association of comorbidity groups by human organ systems with AD dementia prognosis

The mean follow-up time was 2.4 years (SD = 1.9). The number of events, the number of people at risk, and the follow-up time for each transition are reported in Table S3.

Only eye and adnexal diseases, along with certain unspecific healthcare interactions, were associated with cognitive deterioration in non-institutionalized individuals. These diseases were all associated with lower hazards of cognitive deterioration (Table 2, Table S4, and Table S5). In institutionalized individuals, neoplasms were associated with slower progression from very mild to mild AD dementia, diseases of the circulatory system were associated with slower progression from mild to moderate AD dementia, and diseases of the ear and mastoid process were associated with lower hazards of progression from moderate to severe AD dementia in institutionalized individuals (Table 2, Table S4, and Table S5).

**Table 2:** Comorbidity groups by human organ systems significantly associated with AD dementia prognosis

Transition	Predictor	No. events/No. people at risk	HR (95% CI)	C-index
From very mild to mild AD dementia	VII Diseases of the eye and adnexa	539/1263	0.87 (0.77–0.98)	0.536
From very mild AD dementia to institutionalization	V Mental, Behavioral and Neurodevelopmental disorders	60/333	1.86 (1.37–2.52)	0.665
	XIX Injury, poisoning and certain other consequences of external causes	125/826	1.70 (1.28–2.28)	
From mild to moderate AD dementia	XVIII Symptoms, signs and abnormal clinical and laboratory findings, not elsewhere classified	1212/3181	0.90 (0.83-1.00)	0.540
From mild AD dementia to institutionalization	V Mental, Behavioral and Neurodevelopmental disorders	158/751	1.39 (1.16–1.67)	0.656
	XIX Injury, poisoning and certain other consequences of external causes	383/1799	1.38 (1.18–1.62)	
From mild AD dementia to death	IV Endocrine, nutritional and metabolic diseases	80/1004	1.46 (1.06–2.02)	0.732
	V Mental, Behavioral and Neurodevelopmental disorders	56/649	2.20 (1.58–3.05)	
	IX Diseases of the circulatory system	126/1729	1.58 (1.08–2.30)	
From moderate to severe AD dementia	XIX Injury, poisoning and certain other consequences of external causes	370/1639	0.87 (0.76-1.00)	0.568
	XXI Factors influencing health status and contact with health services	698/2883	0.83 (0.69–0.99)	
From moderate AD dementia to institutionalization	I Certain infectious and parasitic diseases	302/755	1.19 (1.04–1.35)	0.624
	V Mental, Behavioral and Neurodevelopmental disorders	357/905	1.31 (1.16–1.48)	
From moderate AD dementia to death	II Neoplasms	102/1026	1.63 (1.23–2.18)	0.759
	III Diseases of the blood and blood-forming organs and certain disorders involving the immune mechanism	38/266	1.68 (1.17–2.43)	
	IX Diseases of the circulatory system	141/1518	1.51 (1.08–2.10)	
From severe AD dementia to institutionalization	VIII Diseases of the ear and mastoid process	147/218	0.79 (0.65–0.95)	0.544
From institutionalized very mild AD dementia to institutionalized mild AD dementia	II Neoplasms	12/92	0.48 (0.23–0.99)	0.592
From institutionalized very mild AD dementia to death	III Diseases of the blood and blood-forming organs and certain disorders involving the immune mechanism	8/33	4.66 (1.68–13.08)	0.679
From institutionalized mild AD dementia to institutionalized moderate AD dementia	IX Diseases of the circulatory system	60/486	0.61 (0.40–0.96)	0.570
From institutionalized mild AD dementia to death	II Neoplasms	37/266	1.73 (1.06–2.83)	0.658
	X Diseases of the respiratory system	31/184	2.10 (1.29–3.40)	
From institutionalized moderate AD dementia to institutionalized severe AD dementia	VIII Diseases of the ear and mastoid process	45/315	0.70 (0.50–0.97)	0.544
From institutionalized moderate AD dementia to death	II Neoplasms	70/491	1.45 (1.05–2.01)	0.635
	VI Diseases of the nervous system	61/396	1.45 (1.04–2.01)	
From institutionalized severe AD dementia to death	IX Diseases of the circulatory system	423/618	1.28 (1.09–1.51)	0.585

Abbreviations: AD = Alzheimer’s disease; CI = confidence interval; HR = hazard ratio

Notes: ICD codes related to dementia (F00, F01, F02, F03, F05, F06, G30, G31, and R41) were excluded from chapters V

The results were derived from age- and sex-adjusted multistate models that included all comorbidities listed in the table as covariates

Several comorbidities were associated with higher hazards of institutionalization and mortality (Table 2). Mental, behavioral, and neurodevelopmental disorders were consistently associated with higher institutionalization hazards (Table 2). Injury, poisoning, and certain other consequences of external causes and infectious and parasitic diseases were also associated with higher institutionalization hazards (Table 2). Endocrine, nutritional, and metabolic diseases, diseases of the blood and blood-forming organs and certain immune disorders, diseases of the circulatory system, diseases of the respiratory system, and neoplasms were associated with higher mortality rates (Table 2).

The C-statistics were below 0.6 for models using comorbidities together to predict cognitive deterioration but were between 0.6 and 0.7 for models predicting institutionalization, except for institutionalization in severe AD dementia (Table 2). Comorbidities were more informative for predicting mortality, especially in non-institutionalized individuals, with C-statistics from the models being over 0.7 (Table 2).

When introducing interaction terms between the comorbidity groups and age and sex, there were weak interaction effects (p-value for interaction < 0.1) between age and diseases of the circulatory system for predicting death in mild AD dementia and between age and mental, behavioral, and neurodevelopmental disorders for predicting institutionalization in moderate AD dementia (Table S6). There was also a weak interaction effect between sex and mental, behavioral, and neurodevelopmental disorders in predicting institutionalization in mild AD dementia (Table S6).

Sensitivity analyses adjusted for CSF Aβ_42_, P-tau_181_, and T-tau did not significantly change the results (Table S7).

### The associations of selected comorbidities with AD dementia prognosis

Among the six selected pre-specified comorbidities, only T2DM was associated with a lower hazard of progressing from very mild to mild AD dementia, and IHD was associated with a higher hazard of progressing from very mild to mild AD dementia in institutionalized individuals (Table 3). Notably, IBD was also associated with a higher hazard of progression from very mild AD dementia to mild AD dementia, although the association was not statistically significant (Table 3).

**Table 3:** The associations between selected comorbidities and AD dementia prognosis

Transition	Predictor	No. events/No. people at risk	HR (95% CI)	P-value
From very mild to mild AD dementia	T2DM	98/241	0.79 (0.64–0.97)	0.027*
	IHD	140/318	1.00 (0.84–1.20)	0.996
	CKD	9/29	0.85 (0.44–1.65)	0.635
	Stroke	64/160	0.82 (0.63–1.06)	0.123
	Depression	143/287	1.05 (0.88–1.25)	0.606
	IBD	13/26	1.46 (0.85–2.53)	0.173
From very mild AD dementia to institutionalization	T2DM	25/168	1.14 (0.74–1.74)	0.566
	IHD	30/208	1.08 (0.72–1.61)	0.707
	CKD	3/23	1.06 (0.34–3.38)	0.913
	Stroke	17/113	1.01 (0.61–1.68)	0.959
	Depression	37/181	1.95 (1.36–2.79)	0.000*
	IBD	3/16	1.65 (0.53–5.19)	0.389
From very mild AD dementia to death	T2DM	5/148	0.73 (0.29–1.85)	0.503
	IHD	14/192	1.80 (0.95–3.41)	0.073
	CKD	3/23	2.97 (0.88-10.00)	0.078
	Stroke	5/101	1.03 (0.40–2.65)	0.958
	Depression	6/150	1.22 (0.51–2.94)	0.655
	IBD	2/15	3.49 (0.82–14.68)	0.090
From mild to moderate AD dementia	T2DM	153/426	0.96 (0.81–1.14)	0.626
	IHD	231/643	0.91 (0.79–1.05)	0.209
	CKD	14/51	0.70 (0.41–1.20)	0.194
	Stroke	94/272	0.91 (0.74–1.13)	0.418
	Depression	216/538	0.91 (0.79–1.06)	0.225
	IBD	34/74	1.06 (0.76–1.50)	0.709
From mild AD dementia to institutionalization	T2DM	95/368	1.43 (1.14–1.78)	0.002*
	IHD	113/525	1.07 (0.87–1.32)	0.504
	CKD	14/51	1.45 (0.85–2.45)	0.178
	Stroke	72/250	1.54 (1.20–1.97)	0.001*
	Depression	76/398	1.11 (0.87–1.41)	0.397
	IBD	10/50	1.16 (0.62–2.17)	0.640
From mild AD dementia to death	T2DM	26/299	1.40 (0.92–2.16)	0.114
	IHD	44/456	1.48 (1.04–2.10)	0.030*
	CKD	10/47	3.19 (1.67–6.11)	0.000*
	Stroke	23/201	1.79 (1.14–2.79)	0.011*
	Depression	30/352	1.93 (1.31–2.89)	0.001*
	IBD	1/41	0.41 (0.06–2.91)	0.370
From moderate to severe AD dementia	T2DM	62/297	0.90 (0.70–1.18)	0.470
	IHD	88/421	0.92 (0.74–1.16)	0.514
	CKD	7/36	0.89 (0.42–1.87)	0.747
	Stroke	36/200	0.91 (0.65–1.28)	0.611
	Depression	93/375	0.84 (0.68–1.05)	0.120
	IBD	10/48	0.75 (0.40–1.40)	0.366
From moderate AD dementia to institutionalization	T2DM	177/412	1.34 (1.13–1.56)	0.001*
	IHD	216/549	0.99 (0.85–1.15)	0.923
	CKD	12/41	0.88 (0.50–1.56)	0.666
	Stroke	112/276	1.14 (0.93–1.38)	0.200
	Depression	194/476	1.23 (1.05–1.43)	0.009*
	IBD	23/61	0.97 (0.64–1.47)	0.892
From moderate AD dementia to death	T2DM	26/261	1.21 (0.79–1.87)	0.373
	IHD	41/374	1.09 (0.76–1.56)	0.639
	CKD	8/37	2.29 (1.12–4.76)	0.024*
	Stroke	18/182	1.17 (0.71–1.91)	0.533
	Depression	22/304	1.16 (0.74–1.83)	0.509
	IBD	3/41	1.16 (0.37–3.65)	0.800
From severe AD dementia to institutionalization	T2DM	67/91	1.00 (0.77–1.30)	0.995
	IHD	75/103	0.89 (0.69–1.13)	0.329
	CKD	10/16	1.40 (0.75–2.65)	0.286
	Stroke	43/53	0.96 (0.70–1.31)	0.795
	Depression	95/139	0.84 (0.67–1.03)	0.096
	IBD	8/14	0.76 (0.37–1.52)	0.430
From severe AD dementia to death	T2DM	18/42	1.36 (0.81–2.29)	0.239
	IHD	23/51	1.20 (0.75–1.94)	0.449
	CKD	1/7	0.58 (0.08–4.15)	0.582
	Stroke	14/24	1.30 (0.74–2.28)	0.355
	Depression	20/64	0.69 (0.43–1.13)	0.139
	IBD	4/10	1.06 (0.38–3.02)	0.904
From institutionalized very mild AD dementia to mild AD dementia	T2DM	4/31	1.20 (0.40–3.65)	0.747
	IHD	11/35	2.20 (1.03–4.73)	0.042*
	CKD	0/3	-	-
	Stroke	1/28	0.19 (0.03–1.44)	0.108
	Depression	10/48	1.30 (0.61–2.78)	0.500
	IBD	0/3	-	-
From institutionalized very mild AD dementia to death	T2DM	3/30	1.09 (0.30–4.06)	0.889
	IHD	3/27	0.97 (0.25–3.75)	0.970
	CKD	1/4	2.44 (0.31–19.38)	0.400
	Stroke	3/30	0.96 (0.27–3.44)	0.950
	Depression	3/41	1.01 (0.28–3.70)	0.982
	IBD	0/3	-	-
From institutionalized mild AD dementia to moderate AD dementia	T2DM	14/107	1.11 (0.62–1.98)	0.734
	IHD	13/120	0.95 (0.52–1.73)	0.859
	CKD	1/18	0.73 (0.10–5.40)	0.754
	Stroke	8/87	0.73 (0.35–1.52)	0.397
	Depression	19/110	0.94 (0.55–1.60)	0.812
	IBD	1/12	0.53 (0.07–3.85)	0.532
From institutionalized mild AD dementia to death	T2DM	11/104	0.81 (0.40–1.61)	0.542
	IHD	27/134	2.29 (1.37–3.84)	0.002*
	CKD	3/20	1.68 (0.51–5.53)	0.394
	Stroke	13/92	1.08 (0.56–2.07)	0.818
	Depression	14/105	1.20 (0.65–2.22)	0.556
	IBD	1/12	0.59 (0.08–4.31)	0.603
From institutionalized moderate AD dementia to severe AD dementia	T2DM	31/196	0.88 (0.60–1.29)	0.521
	IHD	42/226	1.14 (0.81–1.60)	0.450
	CKD	4/16	1.79 (0.65–4.89)	0.260
	Stroke	23/147	0.94 (0.61–1.47)	0.796
	Depression	39/230	0.84 (0.60–1.19)	0.330
	IBD	3/28	0.94 (0.30–2.96)	0.918
From institutionalized moderate AD dementia to death	T2DM	29/194	1.06 (0.69–1.63)	0.791
	IHD	32/216	1.09 (0.72–1.65)	0.686
	CKD	7/19	3.39 (1.48–7.79)	0.004*
	Stroke	19/143	0.92 (0.56–1.53)	0.760
	Depression	27/218	1.17 (0.77–1.80)	0.456
	IBD	1/26	0.69 (0.10–4.95)	0.710
From institutionalized severe AD dementia to death	T2DM	85/117	1.31 (1.03–1.66)	0.028*
	IHD	100/134	1.13 (0.90–1.41)	0.286
	CKD	12/17	1.58 (0.88–2.84)	0.125
	Stroke	57/88	0.89 (0.67–1.17)	0.400
	Depression	89/152	0.91 (0.73–1.14)	0.414
	IBD	7/12	1.30 (0.61–2.77)	0.493

Abbreviations: AD = Alzheimer’s disease; CI = confidence interval; CKD = chronic kidney disease; HR = hazard ratio; IBD = inflammatory bowel disease; IHD = ischemic heart disease; T2DM = type 2 diabetes

Note: The results were derived from age- and sex-adjusted multistate models that included all comorbidities listed in the table as covariates

*P-value < 0.05

The associations of the selected comorbidities with institutionalization and mortality were more pronounced. T2DM, depression, and stroke were linked to higher hazards of institutionalization, while T2DM, IHD, CKD, stroke, and depression were associated with higher mortality hazards (Table 3). IBD was not associated with either institutionalization or mortality (Table 3).

Both IHD and depression showed interaction effects with age and sex in predicting AD prognosis, and there was an interaction effect between T2DM and sex in predicting institutionalization in people with moderate AD dementia (Table S8). The interaction effects with age varied across comorbidities and health outcomes, with some associations appearing stronger in older individuals compared to younger ones (Table S8). In contrast, the interaction effects between these comorbidities and sex were more consistent, suggesting stronger associations in females than in males (Table S8).

**Fig. 3:**
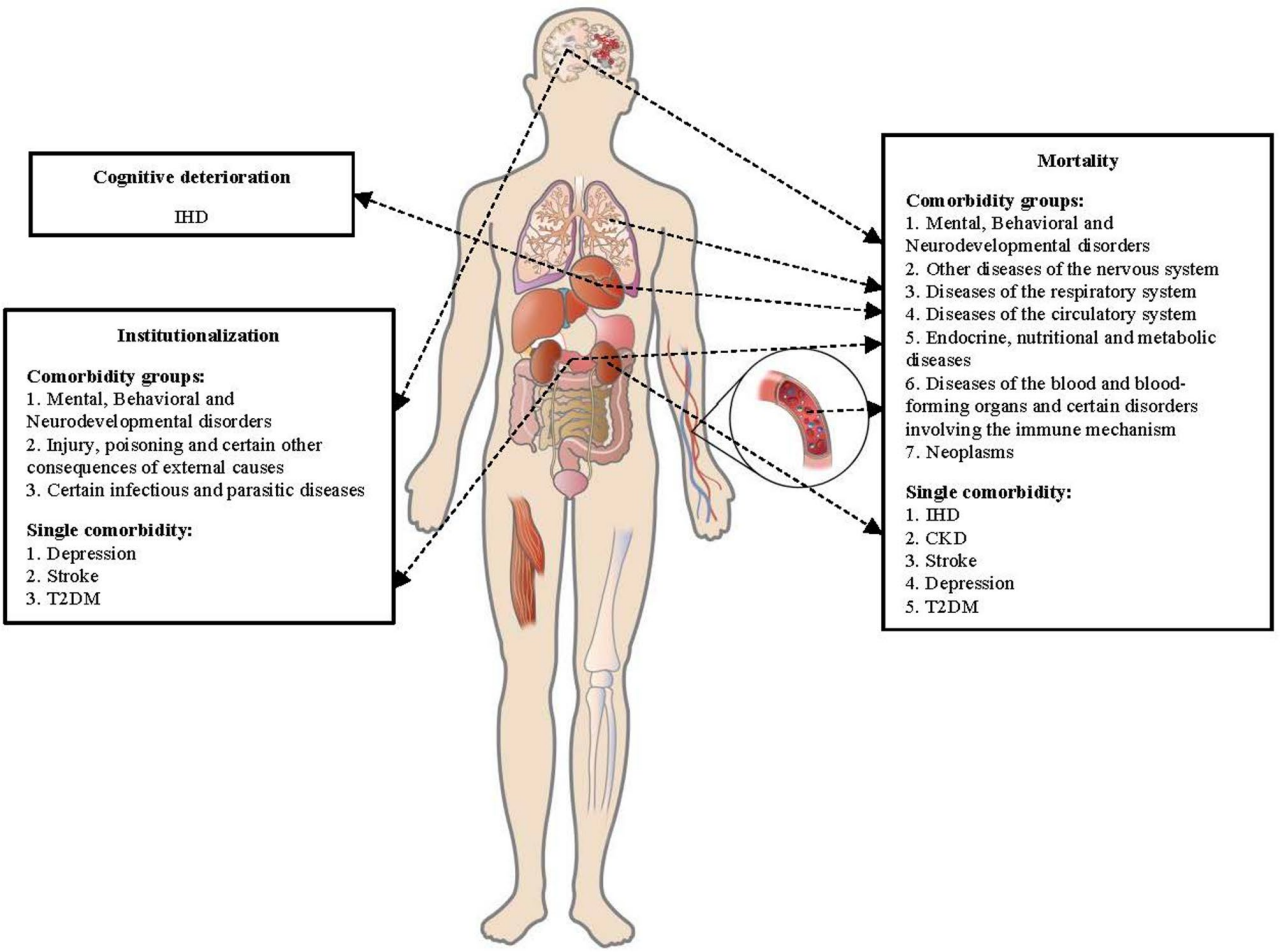
Summary of comorbidities associated with worse prognosis in biomarker-confirmed AD Abbreviations: CKD = chronic kidney disease; IHD = ischemic heart disease; T2DM = type 2 diabetes

The sensitivity analyses showed that the associations of the selected comorbidities with AD prognosis did not change significantly after further adjusting for CSF Aβ42, P-tau181, and T-tau (Table S9). Comorbidities associated with worse prognosis in biomarker-confirmed AD dementia were summarized in Fig. 3.

## Discussion

In this cohort study using Swedish register data, we explored the associations between comorbidities and the prognosis of biomarker-confirmed AD dementia. T2DM was linked to a lower hazard of progressing from very mild to mild AD dementia in non-institutionalized individuals, while IHD was associated with a higher hazard of progression in institutionalized individuals. Mental disorders, including depression, along with T2DM and stroke, were linked to higher hazards of institutionalization. Endocrine-metabolic disorders, such as T2DM, and circulatory system diseases, including IHD and stroke, as well as CKD, were associated with higher mortality rates. Other conditions associated with higher institutionalization hazards included injury, poisoning, and certain other consequences of external causes and infectious and parasitic diseases. Additional conditions associated with higher mortality rates were diseases of the blood and blood-forming organs and certain immune disorders, diseases of the respiratory system, and neoplasms. The observed associations did not differ after adjusting for CSF biomarkers for AD.

The impact of comorbidities on cognitive decline in people with AD dementia is inconsistent in previous research, and the impact specifically in biomarker-confirmed AD dementia has not previously been investigated [11–15]. CCI, a summary comorbidity burden measure that includes T2DM and IHD, has been shown to be associated with faster cognitive decline [11, 14]. Additionally, a study suggested vascular diseases, including T2DM and heart diseases, were associated with faster cognitive decline, although the association was not statistically significant [15]. Our study adds to the literature that in biomarker-confirmed AD dementia, T2DM was associated with a lower hazard of progression from very mild to mild AD dementia, and IHD was associated with a higher hazard of progression from very mild to mild AD dementia. Diabetes being related to slower cognitive decline has been observed previously in people with AD dementia [12, 13]. This contrasts with that, in the general population, diabetes is a well-established risk factor for cognitive impairment and dementia and an important target for dementia prevention [49, 50]. It was hypothesized that AD dementia patients with and without T2DM had different AD neuropathology burdens, with those without T2DM having higher AD neuropathology burdens, which led to faster cognitive decline than AD dementia patients with T2DM [13]. Our sensitivity analyses showed that T2DM was still associated with a lower hazard of progression from very mild to mild AD dementia after adjusting for AD CSF biomarkers. In addition, the impact of diabetes medication may play a role where certain diabetes drugs in patients with T2DM and AD have been found to be associated with slower cognitive decline [51]. Further research is warranted to elucidate the association between T2DM and cognitive decline in people with biomarker-confirmed AD dementia while taking antidiabetic medication into account. It is also noteworthy that IBD was associated with a higher hazard of progression from very mild AD dementia to mild AD dementia, although the association was not statistically significant. Given the close link between the intestinal microenvironment and brain health, possibly through the microbiota-gut-brain axis, the impact of IBD on AD prognosis warrants further investigation [36]. Our study also found that people with diseases of the eye and adnexa, neoplasms, or diseases of the ear and mastoid process had lower hazards of cognitive deterioration. It is unclear whether the associations result from residual confounding (e.g., better access to healthcare resources) or reverse causation (e.g., people with a better prognosis being more likely to receive specialist care for these comorbidities). Future research is needed to elucidate these associations.

Studies investigating the association between comorbidities and future risk of institutionalization in individuals with AD dementia show inconsistent findings. Two studies reported that neither CCI nor Cumulative Illness Rating Scale for Geriatrics (which assesses a broader range of comorbidities than the CCI) were associated with institutionalization risks [20, 25]. In line with these findings, a cross-sectional showed that a higher comorbidity burden, as measured by CCI, was mainly associated with healthcare costs related to medication costs and medical aids rather than formal care services (e.g., home care, institutionalization) [52]. Conversely, another study found that specific conditions such as hip fracture and heart failure were associated with a higher risk of institutionalization [26]. Additionally, the presence of psychiatric symptoms and depression, often denoted as behavioral and psychological symptoms in dementia, has been shown to be a major factor for institutionalization [53]. Our study found several conditions that can predict the future risk of institutionalization in biomarker-confirmed AD dementia, including mental disorders (including depression), T2DM, stroke, and injury. Comorbidities in dementia have been associated with faster functional decline, which can explain the elevated risks of institutionalization in people with biomarker-confirmed AD dementia who have comorbidities [53, 54]. It is noteworthy that CCI does not include mental disorders or injury, which are closely related to decreased functional ability and an increased risk of future institutionalization [55, 56]. Further studies are needed to validate our findings, assess the predictive value of comorbidities for institutionalization risk, and integrate these factors into future prediction models.

Our study also verified the findings in previous research that IHD, stroke, T2DM, CKD, neoplasms, and diseases of the respiratory system were important contributors to elevated mortality rates in people with AD dementia [16–18, 21, 22, 25]. These conditions were also components of existing summary comorbidity burden measures, including CCI, which were associated with higher mortality rates in people with AD dementia [20, 23, 25]. Notably, a previous study based on SveDem data showed that age, male sex, CCI, and living situation were important factors for predicting mortality risks [23]. These studies, together with our studies, highlighted the importance of comorbidities in the prognosis of people with biomarker-confirmed AD dementia. Additionally, our study findings confirmed that commonly used summary comorbidity burden measures, such as CCI, are effective tools for predicting mortality risks in people with AD dementia.

The findings of the present study can help guide the development of prediction models for the prognosis of biomarker-confirmed AD dementia. Incorporating comorbidities into these models is especially important as new disease-modifying treatments emerge. Accurately predicting cognitive decline, functional deterioration, and mortality will not only assist in selecting the right patients for treatment and informing disease prognosis but also enhance the economic evaluations of novel treatments, which often rely on simulations of disease trajectories using patient characteristic information.

The study has several notable strengths. A key strength is its use of a nationwide sample of individuals with clinically diagnosed dementia and presenting specific types of dementia disorders, combined with the availability of CSF biomarkers, which enabled the confirmation of AD pathology in these patients. Additionally, the study benefits from a large sample size and longitudinal cognitive assessments, and it was linked to various Swedish healthcare registers. This linkage allowed for a comprehensive evaluation of the role of comorbidities across the disease trajectory of AD dementia. Furthermore, this study did not choose a specific comorbidity measure or a limited number of diseases. Instead, it explored a broad spectrum of comorbidities, providing valuable insights for future research in selecting significant comorbidities for predicting AD dementia prognosis. However, several limitations of this study should be acknowledged. First, our study population may include people with mixed pathology, as indicated by individuals with FTD in the study population. Second, the identification of comorbidities relied on the Swedish NPR, which lacks primary care data, potentially leading to an underestimation of comorbidity burdens in this study. Future studies could benefit from also incorporating information from primary care and information on medication use to identify comorbidities. Third, cognitive impairment was categorized using MMSE. Although MMSE is commonly used in clinical settings because it is well-known and easily and quickly performed, it has drawbacks, such as ceiling and floor effects, which may not accurately capture cognitive deterioration. This measurement error may have affected our estimation of the associations between comorbidities and cognitive deterioration. Fourthly, our study did not include functional measures such as activities of daily living or other assessments, which, combined with MMSE, could have better differentiated AD stages. Fifthly, the prognosis of AD is complex and can be influenced by many other factors, including educational background, socioeconomic status, and lifestyle factors, which are also closely related to comorbidities [57, 58]. However, this study could not investigate the effects of these factors on the associations of comorbidities with cognitive deterioration, institutionalization, and mortality due to a lack of relevant data. Sixthly, this comprehensive assessment of the associations between comorbidities and the prognosis of biomarker-confirmed AD involved a large number of analyses. Consequently, the findings should be considered hypothesis-generating and interpreted with caution, as they require validation in confirmatory studies due to the increased risk of false-positive results. Lastly, the study population was younger and had fewer comorbidities compared to other individuals in SveDem because younger patients with fewer comorbidities were more often referred to memory clinics to go through extensive assessments, including CSF tests. This may limit the generalizability of the findings to the broader dementia population in Sweden or in other settings.

## Conclusions

In conclusion, this Swedish register-based cohort study identified several comorbid conditions associated with the prognosis of biomarker-confirmed AD dementia. These comorbidities can serve as important predictors of future risks for institutionalization and mortality, highlighting the need for their inclusion in future prognostic prediction models and economic evaluations.

## Electronic supplementary material

Below is the link to the electronic supplementary material.

Supplementary Material 1

## Data Availability

In accordance with European and Swedish regulations, we are unable to share the study data with the public. Researchers may access data from SveDem and other Swedish registers after obtaining ethical permits and permissions from the register holders.

## Abbreviations

AD: Alzheimer’s disease
CCI: Charlson comorbidity index
CI: Confidence interval
CKD: Chronic kidney disease
CSF: Cerebrospinal fluid
FTD: Frontotemporal dementia
HR: Hazard ratio
IBD: Inflammatory bowel disease
ICD-10: The 10th revision of the International Classification of Diseases
IHD: Ischemic heart disease
LBD: Lewy body dementia
MCI: Mild cognitive impairment
MMSE: Mini-Mental State Examination
NPR: The Swedish National Patient Register
T2DM: Type-2 diabetes
SD: Standard deviation
SveDem: The Swedish Register of Cognitive/Dementia Disorders
VaD: Vascular dementia
