# Correction: Comorbidities predict institutionalization and mortality in biomarker-confirmed alzheimer’s disease

**DOI:** 10.1186/s13195-026-02072-x

**Published:** 2026-05-20

**Authors:** Xin Xia, Alice Clark, Niels Juul Brogaard, Alex Mourer, Anna Areovimata, Maria Eriksdotter, Henrik Zetterberg, Silke Kern, Tobias Skillbäck, Linus Jönsson

**Affiliations:** 1 Department of Neurobiology, Care Sciences and Society, Section for Neurogeriatrics, Karolinska Institutet, Solna, Sweden; 2 Novo Nordisk A/S, Bagsvaerd, Denmark; 3 Division of Clinical Geriatrics, Department of Neurobiology, Care Sciences and Society, Karolinska Institutet, Huddinge, Sweden; 4 Theme Inflammation and Aging, Karolinska University Hospital, Huddinge, Sweden; 5 Department of Psychiatry and Neurochemistry, Institute of Neuroscience and Physiology, Sahlgrenska Academy, University of Gothenburg, Göteborg, Sweden; 6 Clinical Neurochemistry Laboratory, Sahlgrenska University Hospital, Göteborg, Sweden; 7 Department of Neurodegenerative Disease, UCL Institute of Neurology, London, UK; 8 UK Dementia Research Institute at UCL, London, UK; 9 Hong Kong Center for Neurodegenerative Diseases, Hong Kong, China; 10 Wisconsin Alzheimer’s Disease Research Center, School of Medicine and Public Health, University of Wisconsin, University of Wisconsin-Madison, Madison, USA; 11 Neuropsychiatric Epidemiology Unit, Department of Psychiatry and Neurochemistry, Sahlgrenska Academy, University of Gothenburg, Göteborg, Sweden; 12 Region Västra Götaland, Department of Neuropsychiatry, Sahlgrenska University Hospital, Göteborg, Sweden; 13 Clinical Dementia Research, Department of Psychiatry and Neurochemistry, Sahlgrenska Academy, University of Gothenburg, Göteborg, Sweden; 14 Karolinska Institutet, Solnavägen 30, floor 10, BioClinicum, Solna, 171 64 Sweden

**Correction** **:** **Alz Res Therapy 17, 155 (2025)**

**https://doi.org/10.1186/s13195-025-01807-6**

Following publication of the original article [1], some numbers under column "No. events/No. people at risk" in Table 3 are shown as months.

The corrected Table 3.

**Table 3:** The associations between selected comorbidities and AD dementia prognosis

Transition	Predictor	No. events/No. people at risk	HR (95% CI)	*P*-value
From very mild to mild AD dementia	T2DM	98/241	0.79 (0.64—0.97)	0.027*
	IHD	140/318	1.00 (0.84—1.20)	0.996
	CKD	9/29	0.85 (0.44—1.65)	0.635
	Stroke	64/160	0.82 (0.63—1.06)	0.123
	Depression	143/287	1.05 (0.88—1.25)	0.606
	IBD	13/26	1.46 (0.85—2.53)	0.173
From very mild AD dementia to institutionalization	T2DM	25/168	1.14 (0.74—1.74)	0.566
	IHD	30/208	1.08 (0.72—1.61)	0.707
	CKD	3/23	1.06 (0.34—3.38)	0.913
	Stroke	17/113	1.01 (0.61—1.68)	0.959
	Depression	37/181	1.95 (1.36—2.79)	0.000*
	IBD	3/16	1.65 (0.53—5.19)	0.389
From very mild AD dementia to death	T2DM	5/148	0.73 (0.29—1.85)	0.503
	IHD	14/192	1.80 (0.95—3.41)	0.073
	CKD	3/23	2.97 (0.88—10.00)	0.078
	Stroke	5/101	1.03 (0.40—2.65)	0.958
	Depression	6/150	1.22 (0.51—2.94)	0.655
	IBD	2/15	3.49 (0.82—14.68)	0.090
From mild to moderate AD dementia	T2DM	153/426	0.96 (0.81—1.14)	0.626
	IHD	231/643	0.91 (0.79—1.05)	0.209
	CKD	14/51	0.70 (0.41—1.20)	0.194
	Stroke	94/272	0.91 (0.74—1.13)	0.418
	Depression	216/538	0.91 (0.79—1.06)	0.225
	IBD	34/74	1.06 (0.76—1.50)	0.709
From mild AD dementia to institutionalization	T2DM	95/368	1.43 (1.14—1.78)	0.002*
	IHD	113/525	1.07 (0.87—1.32)	0.504
	CKD	14/51	1.45 (0.85—2.45)	0.178
	Stroke	72/250	1.54 (1.20—1.97)	0.001*
	Depression	76/398	1.11 (0.87—1.41)	0.397
	IBD	10/50	1.16 (0.62—2.17)	0.640
From mild AD dementia to death	T2DM	26/299	1.40 (0.92—2.16)	0.114
	IHD	44/456	1.48 (1.04—2.10)	0.030*
	CKD	10/47	3.19 (1.67—6.11)	0.000*
	Stroke	23/201	1.79 (1.14—2.79)	0.011*
	Depression	30/352	1.93 (1.31—2.89)	0.001*
	IBD	1/41	0.41 (0.06—2.91)	0.370
From moderate to severe AD dementia	T2DM	62/297	0.90 (0.70—1.18)	0.470
	IHD	88/421	0.92 (0.74—1.16)	0.514
	CKD	7/36	0.89 (0.42—1.87)	0.747
	Stroke	36/200	0.91 (0.65—1.28)	0.611
	Depression	93/375	0.84 (0.68—1.05)	0.120
	IBD	10/48	0.75 (0.40—1.40)	0.366
From moderate AD dementia to institutionalization	T2DM	177/412	1.34 (1.13—1.56)	0.001*
	IHD	216/549	0.99 (0.85—1.15)	0.923
	CKD	12/41	0.88 (0.50—1.56)	0.666
	Stroke	112/276	1.14 (0.93—1.38)	0.200
	Depression	194/476	1.23 (1.05—1.43)	0.009*
	IBD	23/61	0.97 (0.64—1.47)	0.892
From moderate AD dementia to death	T2DM	26/261	1.21 (0.79—1.87)	0.373
	IHD	41/374	1.09 (0.76—1.56)	0.639
	CKD	8/37	2.29 (1.12—4.76)	0.024*
	Stroke	18/182	1.17 (0.71—1.91)	0.533
	Depression	22/304	1.16 (0.74—1.83)	0.509
	IBD	3/41	1.16 (0.37—3.65)	0.800
From severe AD dementia to institutionalization	T2DM	67/91	1.00 (0.77—1.30)	0.995
	IHD	75/103	0.89 (0.69—1.13)	0.329
	CKD	10/16	1.40 (0.75—2.65)	0.286
	Stroke	43/53	0.96 (0.70—1.31)	0.795
	Depression	95/139	0.84 (0.67—1.03)	0.096
	IBD	8/14	0.76 (0.37—1.52)	0.430
From severe AD dementia to death	T2DM	18/42	1.36 (0.81—2.29)	0.239
	IHD	23/51	1.20 (0.75—1.94)	0.449
	CKD	1/7	0.58 (0.08—4.15)	0.582
	Stroke	14/24	1.30 (0.74—2.28)	0.355
	Depression	20/64	0.69 (0.43—1.13)	0.139
	IBD	4/10	1.06 (0.38—3.02)	0.904
From institutionalized very mild AD dementia to mild AD dementia	T2DM	4/31	1.20 (0.40—3.65)	0.747
	IHD	11/35	2.20 (1.03—4.73)	0.042*
	CKD	0/3	-	-
	Stroke	1/28	0.19 (0.03—1.44)	0.108
	Depression	10/48	1.30 (0.61—2.78)	0.500
	IBD	0/3	-	-
From institutionalized very mild AD dementia to death	T2DM	3/30	1.09 (0.30—4.06)	0.889
	IHD	3/27	0.97 (0.25—3.75)	0.970
	CKD	1/4	2.44 (0.31—19.38)	0.400
	Stroke	3/30	0.96 (0.27—3.44)	0.950
	Depression	3/41	1.01 (0.28—3.70)	0.982
	IBD	0/3	-	-
From institutionalized mild AD dementia to moderate AD dementia	T2DM	14/107	1.11 (0.62—1.98)	0.734
	IHD	13/120	0.95 (0.52—1.73)	0.859
	CKD	1/18	0.73 (0.10—5.40)	0.754
	Stroke	8/87	0.73 (0.35—1.52)	0.397
	Depression	19/110	0.94 (0.55—1.60)	0.812
	IBD	1/12	0.53 (0.07—3.85)	0.532
From institutionalized mild AD dementia to death	T2DM	11/104	0.81 (0.40—1.61)	0.542
	IHD	27/134	2.29 (1.37—3.84)	0.002*
	CKD	3/20	1.68 (0.51—5.53)	0.394
	Stroke	13/92	1.08 (0.56—2.07)	0.818
	Depression	14/105	1.20 (0.65—2.22)	0.556
	IBD	1/12	0.59 (0.08—4.31)	0.603
From institutionalized moderate AD dementia to severe AD dementia	T2DM	31/196	0.88 (0.60—1.29)	0.521
	IHD	42/226	1.14 (0.81—1.60)	0.450
	CKD	4/16	1.79 (0.65—4.89)	0.260
	Stroke	23/147	0.94 (0.61—1.47)	0.796
	Depression	39/230	0.84 (0.60—1.19)	0.330
	IBD	3/28	0.94 (0.30—2.96)	0.918
From institutionalized moderate AD dementia to death	T2DM	29/194	1.06 (0.69—1.63)	0.791
	IHD	32/216	1.09 (0.72—1.65)	0.686
	CKD	7/19	3.39 (1.48—7.79)	0.004*
	Stroke	19/143	0.92 (0.56—1.53)	0.760
	Depression	27/218	1.17 (0.77—1.80)	0.456
	IBD	1/26	0.69 (0.10—4.95)	0.710
From institutionalized severe AD dementia to death	T2DM	85/117	1.31 (1.03—1.66)	0.028*
	IHD	100/134	1.13 (0.90—1.41)	0.286
	CKD	12/17	1.58 (0.88—2.84)	0.125
	Stroke	57/88	0.89 (0.67—1.17)	0.400
	Depression	89/152	0.91 (0.73—1.14)	0.414
	IBD	7/12	1.30 (0.61—2.77)	0.493

The results were derived from age- and sex-adjusted multistate models that included all comorbidities listed in the table as covariates

*Abbreviations*: *AD* Alzheimer's disease, *CI* confidence interval, *CKD* chronic kidney disease, *HR* hazard ratio, *IBD* inflammatory bowel disease, *IHD* ischemic heart disease, *T2DM* type 2 diabetes

*P-value < 0.05

The old version of Table 3.

**Table 3:** The associations between selected comorbidities and AD dementia prognosis

Transition	Predictor	No. events/No. people at risk	HR (95% CI)	*P*-value
From very mild to mild AD dementia	T2DM	98/241	0.79 (0.64—0.97)	0.027*
	IHD	140/318	1.00 (0.84—1.20)	0.996
	CKD	29-Sep	0.85 (0.44—1.65)	0.635
	Stroke	64/160	0.82 (0.63—1.06)	0.123
	Depression	143/287	1.05 (0.88—1.25)	0.606
	IBD	13/26	1.46 (0.85—2.53)	0.173
From very mild AD dementia to institutionalization	T2DM	25/168	1.14 (0.74—1.74)	0.566
	IHD	30/208	1.08 (0.72—1.61)	0.707
	CKD	23-Mar	1.06 (0.34—3.38)	0.913
	Stroke	17/113	1.01 (0.61—1.68)	0.959
	Depression	37/181	1.95 (1.36—2.79)	0.000*
	IBD	16-Mar	1.65 (0.53—5.19)	0.389
From very mild AD dementia to death	T2DM	5/148	0.73 (0.29—1.85)	0.503
	IHD	14/192	1.80 (0.95—3.41)	0.073
	CKD	3/23	2.97 (0.88—10.00)	0.078
	Stroke	5/101	1.03 (0.40—2.65)	0.958
	Depression	6/150	1.22 (0.51—2.94)	0.655
	IBD	15-Feb	3.49 (0.82—14.68)	0.090
From mild to moderate AD dementia	T2DM	153/426	0.96 (0.81—1.14)	0.626
	IHD	231/643	0.91 (0.79—1.05)	0.209
	CKD	14/51	0.70 (0.41—1.20)	0.194
	Stroke	94/272	0.91 (0.74—1.13)	0.418
	Depression	216/538	0.91 (0.79—1.06)	0.225
	IBD	34/74	1.06 (0.76—1.50)	0.709
From mild AD dementia to institutionalization	T2DM	95/368	1.43 (1.14—1.78)	0.002*
	IHD	113/525	1.07 (0.87—1.32)	0.504
	CKD	14/51	1.45 (0.85—2.45)	0.178
	Stroke	72/250	1.54 (1.20—1.97)	0.001*
	Depression	76/398	1.11 (0.87—1.41)	0.397
	IBD	Oct-50	1.16 (0.62—2.17)	0.640
From mild AD dementia to death	T2DM	26/299	1.40 (0.92—2.16)	0.114
	IHD	44/456	1.48 (1.04—2.10)	0.030*
	CKD	Oct-47	3.19 (1.67—6.11)	0.000*
	Stroke	23/201	1.79 (1.14—2.79)	0.011*
	Depression	30/352	1.93 (1.31—2.89)	0.001*
	IBD	Jan-41	0.41 (0.06—2.91)	0.370
From moderate to severe AD dementia	T2DM	62/297	0.90 (0.70—1.18)	0.470
	IHD	88/421	0.92 (0.74—1.16)	0.514
	CKD	Jul-36	0.89 (0.42—1.87)	0.747
	Stroke	36/200	0.91 (0.65—1.28)	0.611
	Depression	93/375	0.84 (0.68—1.05)	0.120
	IBD	Oct-48	0.75 (0.40—1.40)	0.366
From moderate AD dementia to institutionalization	T2DM	177/412	1.34 (1.13—1.56)	0.001*
	IHD	216/549	0.99 (0.85—1.15)	0.923
	CKD	Dec-41	0.88 (0.50—1.56)	0.666
	Stroke	112/276	1.14 (0.93—1.38)	0.200
	Depression	194/476	1.23 (1.05—1.43)	0.009*
	IBD	23/61	0.97 (0.64—1.47)	0.892
From moderate AD dementia to death	T2DM	26/261	1.21 (0.79—1.87)	0.373
	IHD	41/374	1.09 (0.76—1.56)	0.639
	CKD	Aug-37	2.29 (1.12—4.76)	0.024*
	Stroke	18/182	1.17 (0.71—1.91)	0.533
	Depression	22/304	1.16 (0.74—1.83)	0.509
	IBD	Mar-41	1.16 (0.37—3.65)	0.800
From severe AD dementia to institutionalization	T2DM	67/91	1.00 (0.77—1.30)	0.995
	IHD	75/103	0.89 (0.69—1.13)	0.329
	CKD	16-Oct	1.40 (0.75—2.65)	0.286
	Stroke	43/53	0.96 (0.70—1.31)	0.795
	Depression	95/139	0.84 (0.67—1.03)	0.096
	IBD	14-Aug	0.76 (0.37—1.52)	0.430
From severe AD dementia to death	T2DM	18/42	1.36 (0.81—2.29)	0.239
	IHD	23/51	1.20 (0.75—1.94)	0.449
	CKD	7-Jan	0.58 (0.08—4.15)	0.582
	Stroke	14/24	1.30 (0.74—2.28)	0.355
	Depression	20/64	0.69 (0.43—1.13)	0.139
	IBD	10-Apr	1.06 (0.38—3.02)	0.904
From institutionalized very mild AD dementia to mild AD dementia	T2DM	Apr-31	1.20 (0.40—3.65)	0.747
	IHD	Nov-35	2.20 (1.03—4.73)	0.042*
	CKD	0/3	-	-
	Stroke	28-Jan	0.19 (0.03—1.44)	0.108
	Depression	Oct-48	1.30 (0.61—2.78)	0.500
	IBD	0/3	-	-
From institutionalized very mild AD dementia to death	T2DM	30-Mar	1.09 (0.30—4.06)	0.889
	IHD	27-Mar	0.97 (0.25—3.75)	0.970
	CKD	4-Jan	2.44 (0.31—19.38)	0.400
	Stroke	30-Mar	0.96 (0.27—3.44)	0.950
	Depression	41-Mar	1.01 (0.28—3.70)	0.982
	IBD	0/3	-	-
From institutionalized mild AD dementia to moderate AD dementia	T2DM	14/107	1.11 (0.62—1.98)	0.734
	IHD	13/120	0.95 (0.52—1.73)	0.859
	CKD	18-Jan	0.73 (0.10—5.40)	0.754
	Stroke	Aug-87	0.73 (0.35—1.52)	0.397
	Depression	19/110	0.94 (0.55—1.60)	0.812
	IBD	12-Jan	0.53 (0.07—3.85)	0.532
From institutionalized mild AD dementia to death	T2DM	11/104	0.81 (0.40—1.61)	0.542
	IHD	27/134	2.29 (1.37—3.84)	0.002*
	CKD	20-Mar	1.68 (0.51—5.53)	0.394
	Stroke	13/92	1.08 (0.56—2.07)	0.818
	Depression	14/105	1.20 (0.65—2.22)	0.556
	IBD	12-Jan	0.59 (0.08—4.31)	0.603
From institutionalized moderate AD dementia to severe AD dementia	T2DM	31/196	0.88 (0.60—1.29)	0.521
	IHD	42/226	1.14 (0.81—1.60)	0.450
	CKD	16-Apr	1.79 (0.65—4.89)	0.260
	Stroke	23/147	0.94 (0.61—1.47)	0.796
	Depression	39/230	0.84 (0.60—1.19)	0.330
	IBD	28-Mar	0.94 (0.30—2.96)	0.918
From institutionalized moderate AD dementia to death	T2DM	29/194	1.06 (0.69—1.63)	0.791
	IHD	32/216	1.09 (0.72—1.65)	0.686
	CKD	19-Jul	3.39 (1.48—7.79)	0.004*
	Stroke	19/143	0.92 (0.56—1.53)	0.760
	Depression	27/218	1.17 (0.77—1.80)	0.456
	IBD	26-Jan	0.69 (0.10—4.95)	0.710
From institutionalized severe AD dementia to death	T2DM	85/117	1.31 (1.03—1.66)	0.028*
	IHD	100/134	1.13 (0.90—1.41)	0.286
	CKD	17-Dec	1.58 (0.88—2.84)	0.125
	Stroke	57/88	0.89 (0.67—1.17)	0.400
	Depression	89/152	0.91 (0.73—1.14)	0.414
	IBD	12-Jul	1.30 (0.61—2.77)	0.493

The results were derived from age- and sex-adjusted multistate models that included all comorbidities listed in the table as covariates

*Abbreviations*: *AD* Alzheimer's disease, *CI* confidence interval, *CKD* chronic kidney disease, *HR* hazard ratio, *IBD* inflammatory bowel disease, *IHD* ischemic heart disease, *T2DM* type 2 diabetes

*P-value < 0.05

The original article [1] has been corrected.
