# Supplementary material for: Comorbidities predict institutionalization and mortality in biomarker-confirmed alzheimer’s disease
Source: Alzheimers Res Ther. 2025 Jul 12;17:155. doi: 10.1186/s13195-025-01807-6 (PMC12255059; doi:10.1186/s13195-025-01807-6)
Supplement: Supplementary file 1 — Supplementary Material 1 [file 13195_2025_1807_MOESM1_ESM.docx]

**Table of contents**

[Methodology for studying the associations of biomarker-confirmed AD dementia with comorbidities with time-varying AD dementia stages 2](#_Toc197429122)

[Figure S1. The associations of biomarker-confirmed AD dementia with comorbidities with time-varying AD dementia stages 3](#_Toc197429123)

[Figure S2. The associations of biomarker-confirmed AD dementia with selected comorbidities with time-varying AD dementia stages 4](#_Toc197429124)

[Table S1. Characteristics at index dates of individuals from SveDem included and excluded from the study 5](#_Toc197429125)

[Table S2. Frequency of comorbidities in patients with different severities of biomarker-confirmed AD dementia at index dates 8](#_Toc197429126)

[Table S3. The number of events, the number of people at risk, and the follow-up time for each transition in multistate models 10](#_Toc197429127)

[Table S4. Hazard ratios for individual ICD-10 chapters in separate multistate models 11](#_Toc197429128)

[Table S5. Hazard ratios for significant ICD-10 chapters simultaneously included in one multistate model 27](#_Toc197429129)

[Table S6. Interactions between comorbidity groups associated with AD dementia prognosis and age and sex 30](#_Toc197429130)

[Table S7. Comorbidity groups by human organ systems significantly associated with AD dementia prognosis, adjusted for CSF biomarkers 32](#_Toc197429131)

[Table S8. Interactions between selected comorbidities and age and sex 34](#_Toc197429132)

[Table S9. The associations between selected comorbidities and AD dementia prognosis, adjusted for CSF biomarkers 36](#_Toc197429133)

# Methodology for studying the associations of biomarker-confirmed AD dementia with comorbidities with time-varying AD dementia stages

These preliminary analyses investigated the occurrence of comorbidities across different stages of AD dementia while accounting for the effects of age and sex. The analytical sample included individuals with AD dementia, each matched by age and sex to two individuals without dementia. Associations between time-varying AD dementia stages and comorbidities were examined using stratified Cox proportional hazard regressions, with separate models for each ICD-10 chapter-based comorbidity group and pre-specified comorbidities. The follow-up period began 5 years prior to the index date (the date of dementia diagnosis minus 5 years, referred to as Pre-diagnostic). Stages of MCI and AD dementia (Pre-diagnostic, MCI, very mild AD dementia, mild AD dementia, moderate AD dementia, and severe AD dementia) were treated as time-dependent covariates.


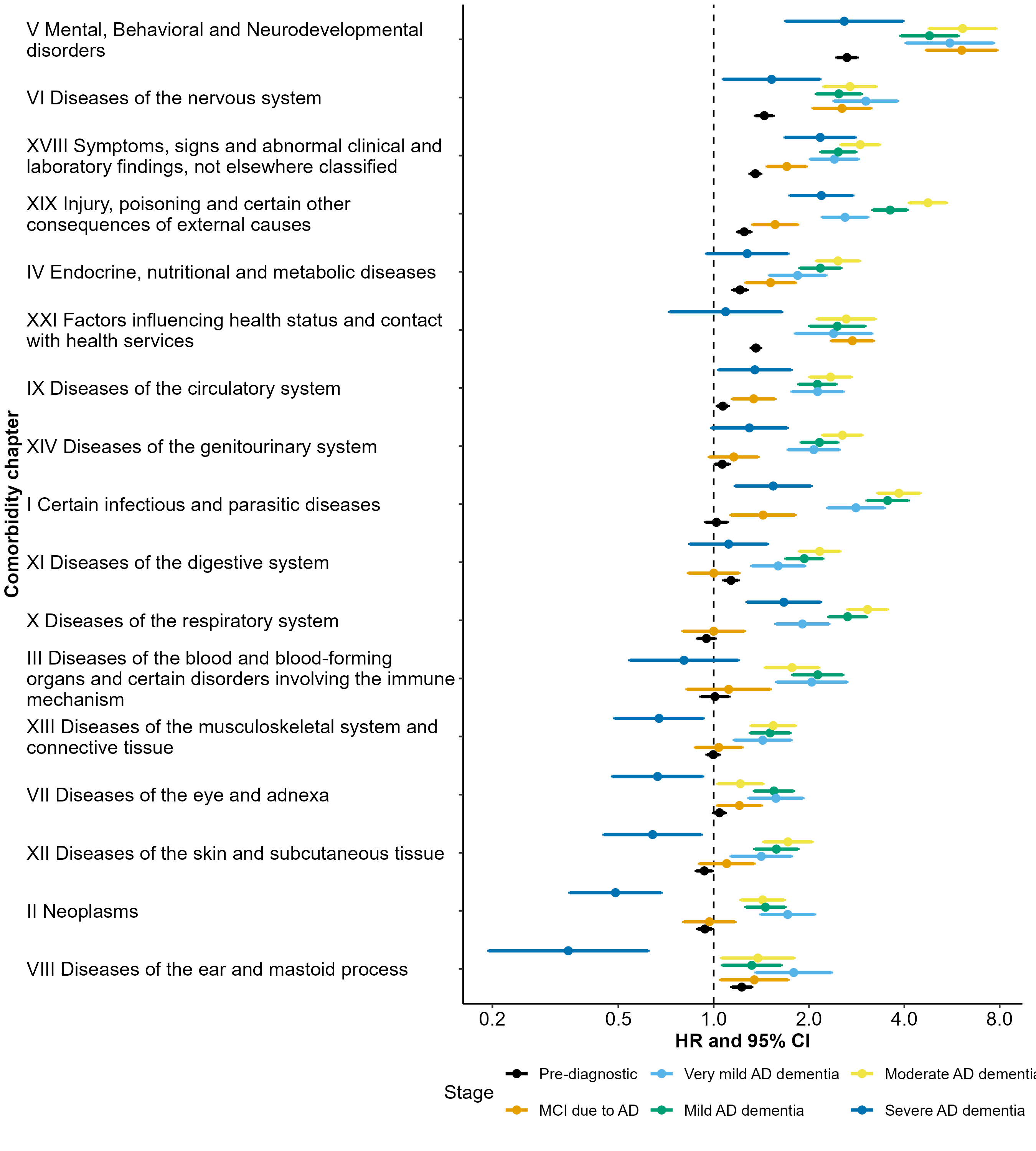


# Figure S1. The associations of biomarker-confirmed AD dementia with comorbidities with time-varying AD dementia stages

Abbreviations: AD = Alzheimer’s disease; CI = confidence interval; HR = hazard ratio.

Notes: ICD codes related to dementia (F00, F01, F02, F03, F05, F06, G30, G31, and R41) were excluded from chapters V, VI, and XVIII. All HRs are from stratified Cox regressions with age- and sex- matched controls as the reference group.


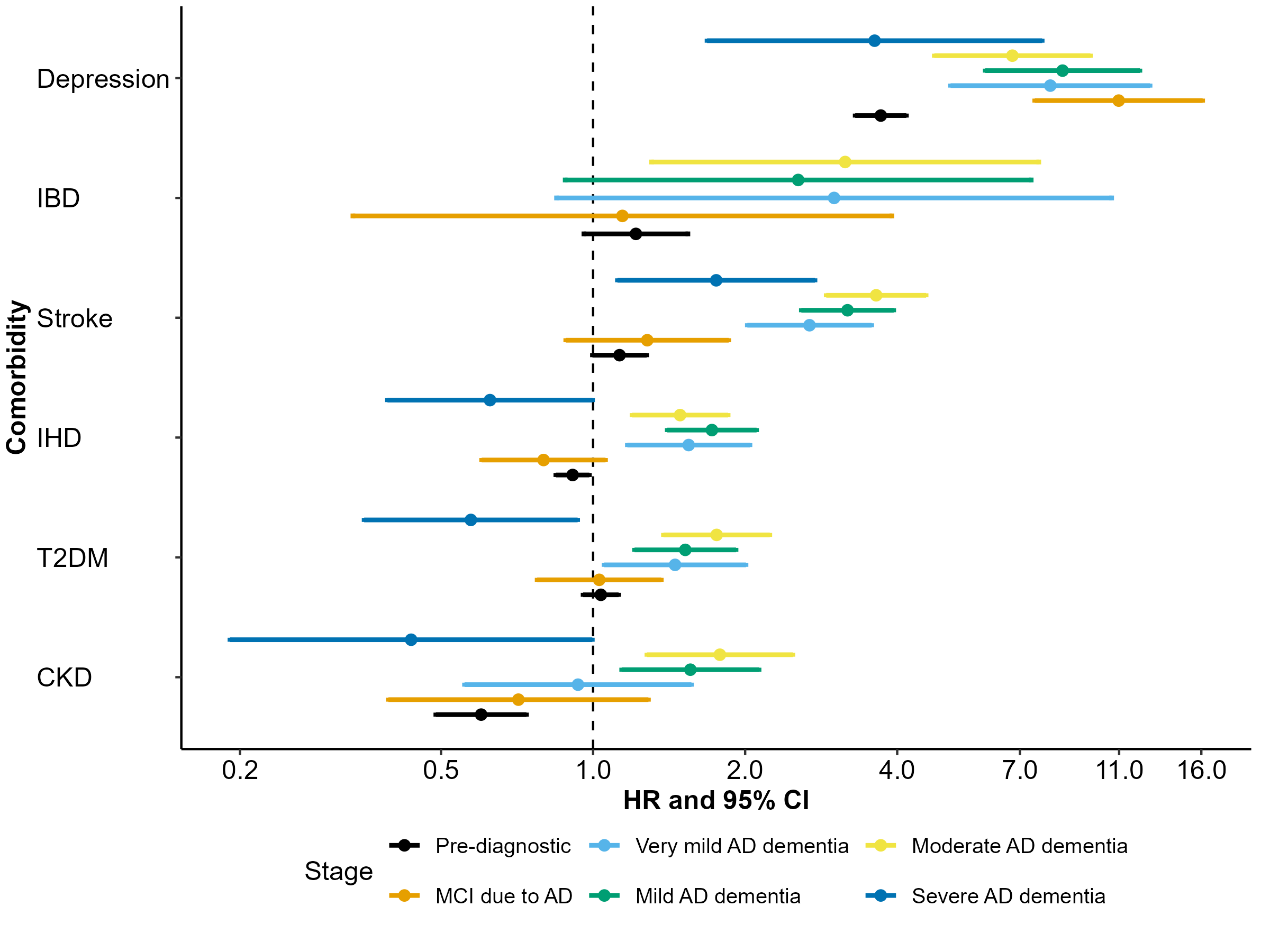


# Figure S2. The associations of biomarker-confirmed AD dementia with selected comorbidities with time-varying AD dementia stages

AD = Alzheimer’s disease; CI = confidence interval; CKD = chronic kidney disease; HR = hazard ratio; IBD = inflammatory bowel disease; IHD = ischemic heart disease; T2DM = type 2 diabetes. HR for the association between severe AD dementia and IBD was not reported due to too few events. All HRs are from stratified Cox regressions with age- and sex- matched controls as the reference group.

# Table S1. Characteristics at index dates of individuals from SveDem included and excluded from the study

|  | **Included (N=10857)** | **Excluded due to missing CSF Aβ status (N=81873)** | **Excluded due to CSF Aβ-negative (N=2803)** | **Excluded due to missing MMSE (N=207)** | **Overall (N=95740)** |
| --- | --- | --- | --- | --- | --- |
| **Age (years), mean (SD)** | 73.85 (7.74) | 81.14 (7.38) | 72.02 (8.38) | 73.65 (8.47) | 80.03 (7.94) |
| **Sex-female, n (%)** | 6052 (55.74%) | 48391 (59.10%) | 1156 (41.24%) | 109 (52.66%) | 55708 (58.19%) |
| **AD dementia stage, n (%)** |  |  |  |  |  |
| Very mild AD dementia | 2809 (25.87%) | 12406 (15.15%) | 779 (27.79%) | - | 15994 (16.71%) |
| Mild AD dementia | 4507 (41.51%) | 30712 (37.51%) | 1143 (40.78%) | - | 36362 (37.98%) |
| Moderate AD dementia | 3084 (28.41%) | 30418 (37.15%) | 666 (23.76%) | - | 34168 (35.69%) |
| Severe AD dementia | 349 (3.21%) | 5035 (6.15%) | 84 (3.00%) | - | 5468 (5.71%) |
| Missing | 108 (1.0%) | 3302 (4.0%) | 131 (4.7%) | 207 (100%) | 3748 (3.9%) |
| **Diagnostic setting, n (%)** |  |  |  |  |  |
| Primary care | 1610 (14.83%) | 41359 (50.52%) | 601 (21.44%) | 69 (33.33%) | 43639 (45.58%) |
| Specialist care | 9247 (85.17%) | 40514 (49.48%) | 2202 (78.56%) | 138 (66.67%) | 52101 (54.42%) |
| **Clinical diagnosis, n (%)** |  |  |  |  |  |
| Late-onset AD | 4725 (43.52%) | 22464 (27.44%) | 290 (10.35%) | 62 (29.95%) | 27541 (28.77%) |
| Mixed AD with VaD | 1773 (16.33%) | 15866 (19.38%) | 215 (7.67%) | 16 (7.73%) | 17870 (18.67%) |
| Early-onset AD | 1507 (13.88%) | 1639 (2.00%) | 64 (2.28%) | 37 (17.87%) | 3247 (3.39%) |
| VaD | 820 (7.55%) | 16630 (20.31%) | 700 (24.97%) | 20 (9.66%) | 18170 (18.98%) |
| LBD | 328 (3.02%) | 1534 (1.87%) | 206 (7.35%) | 16 (7.73%) | 2084 (2.18%) |
| FTD | 217 (2.00%) | 932 (1.14%) | 367 (13.09%) | 5 (2.42%) | 1521 (1.59%) |
| PDD | 77 (0.71%) | 1248 (1.52%) | 105 (3.75%) | 4 (1.93%) | 1434 (1.50%) |
| Other dementia | 202 (1.86%) | 2144 (2.62%) | 185 (6.60%) | 6 (2.90%) | 2537 (2.65%) |
| Unspecified dementia | 1173 (10.80%) | 19345 (23.63%) | 661 (23.58%) | 38 (18.36%) | 21217 (22.16%) |
| MCI | 35 (0.32%) | 71 (0.09%) | 10 (0.36%) | 3 (1.45%) | 119 (0.12%) |
| **Living arrangement, n (%)** |  |  |  |  |  |
| Institutionalized | 222 (2.04%) | 5519 (6.74%) | 100 (3.57%) | 19 (9.18%) | 5860 (6.12%) |
| Non-institutionalized | 10635 (97.96%) | 76354 (93.26%) | 2703 (96.43%) | 188 (90.82%) | 89880 (93.88%) |
| **Selected comorbidities, n (%)** |  |  |  |  |  |
| T2DM | 988 (9.10%) | 10745 (13.12%) | 445 (15.88%) | 19 (9.18%) | 12197 (12.74%) |
| IHD | 1068 (9.84%) | 12728 (15.55%) | 314 (11.20%) | 26 (12.56%) | 14136 (14.76%) |
| CKD | 133 (1.23%) | 2440 (2.98%) | 59 (2.10%) | 5 (2.42%) | 2637 (2.75%) |
| Stroke | 444 (4.09%) | 7010 (8.56%) | 207 (7.38%) | 17 (8.21%) | 7678 (8.02%) |
| Depression | 985 (9.07%) | 5010 (6.12%) | 371 (13.24%) | 18 (8.70%) | 6384 (6.67%) |
| IBD | 114 (1.05%) | 583 (0.71%) | 30 (1.07%) | 1 (0.48%) | 728 (0.76%) |
| **Anticoagulant or antiplatelet prescription, n (%)** | 3343 (30.79%) | 36518 (44.60%) | 955 (34.07%) | 71 (34.30%) | 40887 (42.71%) |
| **Aβ₄₂/Aβ₄₀ ratio** |  |  |  |  |  |
| Mean (SD) | 0.05 (0.01) | 0.08 (0.02) | 0.09 (0.02) | 0.05 (0.01) | 0.06 (0.02) |
| Missing | 7893 (72.7%) | 81863 (100.0%) | 2004 (71.5%) | 151 (72.9%) | 91911 (96.0%) |
| **Aβ₄₂/P-tau₁₈₁ ratio** |  |  |  |  |  |
| Mean (SD) | 6.59 (3.15) | - | 22.62 (6.08) | 6.83 (3.12) | 9.84 (7.53) |
| Missing | 0 (0%) | 81873 (100%) | 0 (0%) | 0 (0%) | 81873 (85.5%) |

Abbreviations: AD = Alzheimer’s disease; CKD = chronic kidney disease; CSF = cerebrospinal fluid; FTD = Frontotemporal dementia; IBD = inflammatory bowel disease; IHD = ischemic heart disease; LBD = Lewy body dementia; MCI = mild cognitive impairment; MMSE = mini-mental state examination; PDD = Parkinson's disease dementia; SveDem = the Swedish Register of Cognitive Disorders; T2DM = type 2 diabetes; SD = standard deviation; VaD = vascular dementia.

Note: Anticoagulant and antiplatelet use within 3 months prior to index dates was identified from the Swedish National Prescribed Drug Register with Anatomical Therapeutic Chemical (ATC) Classification codes: B01AA for Vitamin K antagonists, B01AE for direct thrombin inhibitors, B01AF for direct factor Xa inhibitors, and B01AC for platelet aggregation inhibitors.

# Table S2. Frequency of comorbidities in patients with different severities of biomarker-confirmed AD dementia at index dates

| ICD-10 and Comorbidity | Very mild AD dementia | Mild AD dementia | Moderate AD dementia | Severe AD dementia |
| --- | --- | --- | --- | --- |
|  | (N = 2809) | (N = 4507) | (N = 3084) | (N = 349) |
| I Certain infectious and parasitic diseases (A00-B99), n (%) | 262 (9.33) | 414 (9.19) | 332 (10.77) | 41 (11.75) |
| II Neoplasms (C00-D48), n (%) | 714 (25.42) | 979 (21.72) | 613 (19.88) | 61 (17.48) |
| III Diseases of the blood and blood-forming organs and certain disorders involving the immune mechanism (D50-D89), n (%) | 134 (4.77) | 254 (5.64) | 222 (7.20) | 18 (5.16) |
| IV Endocrine, nutritional and metabolic diseases (E00-E90), n (%) | 669 (23.82) | 1098 (24.36) | 817 (26.49) | 82 (23.50) |
| T2DM, n (%) | 234 (8.33) | 391 (8.68) | 316 (10.25) | 39 (11.17) |
| V Mental, Behavioral and Neurodevelopmental disorders (F00-F99), n (%) | 484 (17.23) | 736 (16.33) | 569 (18.45) | 73 (20.92) |
| Depression, n (%) | 279 (9.93) | 406 (9.01) | 258 (8.37) | 32 (9.17) |
| VI Diseases of the nervous system (G00-G99), n (%) | 476 (16.95) | 776 (17.22) | 529 (17.15) | 71 (20.34) |
| VII Diseases of the eye and adnexa (H00-H59), n (%) | 1038 (36.95) | 1617 (35.88) | 1112 (36.06) | 111 (31.81) |
| VIII Diseases of the ear and mastoid process (H60-H95), n (%) | 381 (13.56) | 535 (11.87) | 363 (11.77) | 41 (11.75) |
| IX Diseases of the circulatory system (I00-I99), n (%) | 1207 (42.97) | 1977 (43.87) | 1375 (44.58) | 138 (39.54) |
| IHD, n (%) | 273 (9.72) | 446 (9.90) | 320 (10.38) | 21 (6.02) |
| Stroke, n (%) | 110 (3.92) | 178 (3.95) | 132 (4.28) | 17 (4.87) |
| X Diseases of the respiratory system (J00-J99), n (%) | 373 (13.28) | 631 (14.00) | 437 (14.17) | 47 (13.47) |
| XI Diseases of the digestive system (K00-K93), n (%) | 709 (25.24) | 1068 (23.70) | 695 (22.54) | 75 (21.49) |
| IBD, n (%) | 22 (0.78) | 56 (1.24) | 33 (1.07) | 3 (0.86) |
| XII Diseases of the skin and subcutaneous tissue (L00-L99), n (%) | 577 (20.54) | 779 (17.28) | 495 (16.05) | 43 (12.32) |
| XIII Diseases of the musculoskeletal system and connective tissue (M00-M99), n (%) | 945 (33.64) | 1398 (31.02) | 943 (30.58) | 96 (27.51) |
| XIV Diseases of the genitourinary system (N00-N99), n (%) | 722 (25.70) | 1105 (24.52) | 736 (23.87) | 101 (28.94) |
| CKD, n (%) | 35 (1.25) | 58 (1.29) | 34 (1.10) | 6 (1.72) |
| XVII Congenital malformations, deformations and chromosomal abnormalities (Q00-Q99), n (%) | 18 (0.64) | 30 (0.67) | 25 (0.81) | 4 (1.15) |
| XVIII Symptoms, signs and abnormal clinical and laboratory findings, not elsewhere classified (R00-R99), n (%) | 1390 (49.48) | 2237 (49.63) | 1548 (50.19) | 171 (49.00) |
| XIX Injury, poisoning and certain other consequences of external causes (S00-T98), n (%) | 811 (28.87) | 1292 (28.67) | 923 (29.93) | 108 (30.95) |
| XXI Factors influencing health status and contact with health services (Z00-Z99), n (%) | 2176 (77.47) | 3325 (73.77) | 2212 (71.73) | 233 (66.76) |
| XXII Codes for special purposes (U00-U99), n (%) | 28 (1.00) | 58 (1.29) | 52 (1.69) | 6 (1.72) |

Abbreviations: AD = Alzheimer’s disease; CKD = chronic kidney disease; IBD = inflammatory bowel disease; ICD = the 10th revision of the International Classification of Diseases; IHD = ischemic heart disease; T2DM = type 2 diabetes.

Notes: ICD codes related to dementia (F00, F01, F02, F03, F05, F06, G30, G31, and R41) were excluded from chapters V, VI, and XVIII.

The following ICD chapters were excluded due to the few numbers of people with these conditions or lack of data in this study: XV (Pregnancy, childbirth and the puerperium), XVI (Certain conditions originating in the perinatal period), and XX (External causes of morbidity).

# Table S3. The number of events, the number of people at risk, and the follow-up time for each transition in multistate models

| From | To | No. events/No. people at risk | Mean follow-up time (years) |
| --- | --- | --- | --- |
| Very mild AD dementia | Mild AD dementia | 1185/2799 | 1.41 (SD = 1.15) |
|  | Very mild AD dementia +institutionalization | 198/2799 | 1.50 (SD = 1.14) |
|  | Mild AD dementia +institutionalization | — | — |
|  | Death | 49/2799 | 1.04 (SD = 0.83) |
| Mild AD dementia | Moderate AD dementia | 1947/5667 | 1.53 (SD = 1.17) |
|  | Mild AD dementia +institutionalization | 654/5667 | 1.56 (SD = 1.12) |
|  | Moderate AD dementia +institutionalization | — | — |
|  | Death | 168/5667 | 1.01 (SD = 0.96) |
| Moderate AD dementia | Severe AD dementia | 851/4969 | 1.76 (SD = 1.26) |
|  | Moderate AD dementia +institutionalization | 1373/4969 | 1.45 (SD = 0.98) |
|  | Severe AD dementia +institutionalization | — | — |
|  | Death | 192/4969 | 1.19 (SD = 1.03) |
| Severe AD dementia | Severe AD dementia +institutionalization | 743/1174 | 1.63 (SD = 1.52) |
|  | Death | 146/1174 | 1.72 (SD = 1.36) |
| Very mild AD dementia +  Institutionalization | Mild AD dementia +institutionalization | 35/222 | 0.66 (SD = 0.65) |
|  | Death | 17/222 | 0.45 (SD = 0.34) |
| Mild AD dementia +  Institutionalization | Moderate AD dementia +institutionalization | 93/740 | 0.72 (SD = 0.58) |
|  | Death | 68/740 | 0.64 (SD = 0.74) |
| Moderate AD dementia +  Institutionalization | Severe AD dementia +institutionalization | 256/1570 | 0.82 (SD = 0.65) |
|  | Death | 151/1570 | 0.58 (SD = 0.54) |
| Severe AD dementia +Institutionalization | Death | 667/1051 | 2.10 (SD = 1.62) |

Abbreviations: AD = Alzheimer’s disease; SD = standard deviation.

# Table S4. Hazard ratios for individual ICD-10 chapters in separate multistate models

| Transition | Predictor | Biomarker-confirmed AD dementia | | |
| --- | --- | --- | --- | --- |
|  |  | No. events/No. people at risk | HR (95% CI) | P-value |
| From very mild to mild AD dementia | I Certain infectious and parasitic diseases | 199/446 | 0.99 (0.85-1.15) | 0.913 |
|  | II Neoplasms | 436/1010 | 0.91 (0.81-1.03) | 0.141 |
|  | III Diseases of the blood and blood-forming organs and certain disorders involving the immune mechanism | 74/179 | 1.00 (0.79-1.27) | 0.975 |
|  | IV Endocrine, nutritional and metabolic diseases | 339/756 | 0.95 (0.84-1.08) | 0.453 |
|  | T2DM | 98/241 | 0.77 (0.63-0.95) | 0.015* |
|  | V Mental, Behavioral and Neurodevelopmental disorders | 235/508 | 1.04 (0.90-1.21) | 0.558 |
|  | Depression | 143/287 | 1.04 (0.88-1.25) | 0.628 |
|  | VI Diseases of the nervous system | 269/658 | 0.97 (0.85-1.11) | 0.678 |
|  | VII Diseases of the eye and adnexa | 539/1263 | 0.87 (0.77-0.98) | 0.020* |
|  | VIII Diseases of the ear and mastoid process | 276/616 | 0.98 (0.86-1.13) | 0.815 |
|  | IX Diseases of the circulatory system | 603/1341 | 0.96 (0.85-1.08) | 0.492 |
|  | IHD | 140/318 | 0.98 (0.82-1.17) | 0.807 |
|  | Stroke | 64/160 | 0.80 (0.62-1.03) | 0.083 |
|  | X Diseases of the respiratory system | 223/550 | 0.86 (0.75-1.00) | 0.046* |
|  | XI Diseases of the digestive system | 498/1080 | 1.02 (0.90-1.14) | 0.780 |
|  | IBD | 13/26 | 1.46 (0.85-2.53) | 0.172 |
|  | XII Diseases of the skin and subcutaneous tissue | 390/862 | 0.93 (0.82-1.05) | 0.241 |
|  | XIII Diseases of the musculoskeletal system and connective tissue | 585/1304 | 0.97 (0.86-1.08) | 0.568 |
|  | XIV Diseases of the genitourinary system | 500/1123 | 0.91 (0.81-1.03) | 0.125 |
|  | CKD | 9/29 | 0.84 (0.43-1.61) | 0.593 |
|  | XVIII Symptoms, signs and abnormal clinical and laboratory findings, not elsewhere classified | 747/1684 | 0.93 (0.83-1.05) | 0.264 |
|  | XIX Injury, poisoning and certain other consequences of external causes | 516/1217 | 0.93 (0.82-1.04) | 0.187 |
|  | XXI Factors influencing health status and contact with health services | 1009/2223 | 0.85 (0.73-1.00) | 0.050* |
| From very mild AD dementia to institutionalization | I Certain infectious and parasitic diseases | 43/290 | 1.32 (0.94-1.85) | 0.107 |
|  | II Neoplasms | 85/659 | 1.01 (0.76-1.34) | 0.951 |
|  | III Diseases of the blood and blood-forming organs and certain disorders involving the immune mechanism | 20/125 | 1.49 (0.93-2.37) | 0.094 |
|  | IV Endocrine, nutritional and metabolic diseases | 66/483 | 1.08 (0.80-1.45) | 0.631 |
|  | T2DM | 25/168 | 1.16 (0.76-1.76) | 0.500 |
|  | V Mental, Behavioral and Neurodevelopmental disorders | 60/333 | 1.93 (1.42-2.62) | <0.0001* |
|  | Depression | 37/181 | 1.95 (1.36-2.80) | 0.000* |
|  | VI Diseases of the nervous system | 58/447 | 1.16 (0.85-1.57) | 0.355 |
|  | VII Diseases of the eye and adnexa | 111/835 | 0.95 (0.71-1.27) | 0.742 |
|  | VIII Diseases of the ear and mastoid process | 49/389 | 0.92 (0.67-1.28) | 0.637 |
|  | IX Diseases of the circulatory system | 123/861 | 1.22 (0.91-1.64) | 0.183 |
|  | IHD |  |  |  |
|  | Stroke | 17/113 | 1.08 (0.66-1.78) | 0.761 |
|  | X Diseases of the respiratory system | 41/368 | 0.84 (0.59-1.18) | 0.317 |
|  | XI Diseases of the digestive system | 83/665 | 0.95 (0.72-1.27) | 0.733 |
|  | IBD | 3/16 | 1.64 (0.52-5.13) | 0.396 |
|  | XII Diseases of the skin and subcutaneous tissue | 72/544 | 0.97 (0.72-1.30) | 0.833 |
|  | XIII Diseases of the musculoskeletal system and connective tissue | 112/831 | 1.15 (0.86-1.52) | 0.341 |
|  | XIV Diseases of the genitourinary system | 95/718 | 1.00 (0.76-1.32) | 0.994 |
|  | CKD | 3/23 | 1.20 (0.38-3.77) | 0.756 |
|  | XVIII Symptoms, signs and abnormal clinical and laboratory findings, not elsewhere classified | 144/1081 | 1.27 (0.93-1.73) | 0.139 |
|  | XIX Injury, poisoning and certain other consequences of external causes | 125/826 | 1.76 (1.32-2.36) | 0.000* |
|  | XXI Factors influencing health status and contact with health services | 176/1390 | 0.97 (0.63-1.52) | 0.910 |
| From very mild AD dementia to death | I Certain infectious and parasitic diseases | 15/262 | 2.00 (1.08-3.68) | 0.026* |
|  | II Neoplasms | 20/594 | 0.87 (0.49-1.55) | 0.644 |
|  | III Diseases of the blood and blood-forming organs and certain disorders involving the immune mechanism | 4/109 | 1.20 (0.43-3.33) | 0.730 |
|  | IV Endocrine, nutritional and metabolic diseases | 20/437 | 1.43 (0.81-2.53) | 0.220 |
|  | T2DM | 5/148 | 0.74 (0.29-1.88) | 0.533 |
|  | V Mental, Behavioral and Neurodevelopmental disorders | 10/283 | 1.33 (0.66-2.69) | 0.421 |
|  | Depression | 6/150 | 1.40 (0.59-3.30) | 0.445 |
|  | VI Diseases of the nervous system | 14/403 | 1.01 (0.54-1.89) | 0.968 |
|  | VII Diseases of the eye and adnexa | 29/753 | 1.15 (0.64-2.07) | 0.635 |
|  | VIII Diseases of the ear and mastoid process | 13/353 | 1.15 (0.61-2.18) | 0.662 |
|  | IX Diseases of the circulatory system | 37/775 | 2.08 (1.07-4.04) | 0.031* |
|  | IHD | 14/192 | 1.88 (1.00-3.54) | 0.050 |
|  | Stroke | 5/101 | 1.15 (0.46-2.92) | 0.765 |
|  | X Diseases of the respiratory system | 19/346 | 1.85 (1.04-3.29) | 0.036* |
|  | XI Diseases of the digestive system | 20/602 | 0.82 (0.47-1.46) | 0.510 |
|  | IBD | 2/15 | 4.40 (1.06-18.17) | 0.041* |
|  | XII Diseases of the skin and subcutaneous tissue | 17/489 | 0.94 (0.52-1.69) | 0.827 |
|  | XIII Diseases of the musculoskeletal system and connective tissue | 21/740 | 0.78 (0.44-1.37) | 0.382 |
|  | XIV Diseases of the genitourinary system | 24/647 | 1.12 (0.64-1.97) | 0.688 |
|  | CKD | 3/23 | 3.63 (1.12-11.78) | 0.032* |
|  | XVIII Symptoms, signs and abnormal clinical and laboratory findings, not elsewhere classified | 39/976 | 1.83 (0.91-3.66) | 0.090 |
|  | XIX Injury, poisoning and certain other consequences of external causes | 28/729 | 1.44 (0.82-2.55) | 0.205 |
|  | XXI Factors influencing health status and contact with health services | 45/1259 | 1.30 (0.47-3.61) | 0.619 |
| From mild to moderate AD dementia | I Certain infectious and parasitic diseases | 296/821 | 0.97 (0.85-1.10) | 0.607 |
|  | II Neoplasms | 687/1760 | 0.99 (0.90-1.09) | 0.806 |
|  | III Diseases of the blood and blood-forming organs and certain disorders involving the immune mechanism | 128/373 | 0.93 (0.77-1.11) | 0.411 |
|  | IV Endocrine, nutritional and metabolic diseases | 535/1459 | 0.94 (0.85-1.04) | 0.212 |
|  | T2DM | 153/426 | 0.92 (0.78-1.09) | 0.335 |
|  | V Mental, Behavioral and Neurodevelopmental disorders | 362/955 | 0.89 (0.80-1.00) | 0.055 |
|  | Depression | 216/538 | 0.91 (0.79-1.05) | 0.213 |
|  | VI Diseases of the nervous system | 452/1221 | 0.95 (0.85-1.05) | 0.321 |
|  | VII Diseases of the eye and adnexa | 861/2350 | 0.94 (0.86-1.03) | 0.206 |
|  | VIII Diseases of the ear and mastoid process | 403/1095 | 0.96 (0.86-1.07) | 0.452 |
|  | IX Diseases of the circulatory system | 1013/2616 | 1.00 (0.91-1.09) | 0.964 |
|  | IHD | 231/643 | 0.91 (0.79-1.04) | 0.164 |
|  | Stroke | 94/272 | 0.91 (0.74-1.12) | 0.361 |
|  | X Diseases of the respiratory system | 394/1064 | 0.96 (0.86-1.08) | 0.506 |
|  | XI Diseases of the digestive system | 790/2002 | 1.05 (0.96-1.15) | 0.299 |
|  | IBD | 34/74 | 1.07 (0.76-1.50) | 0.691 |
|  | XII Diseases of the skin and subcutaneous tissue | 583/1517 | 0.97 (0.88-1.07) | 0.491 |
|  | XIII Diseases of the musculoskeletal system and connective tissue | 908/2380 | 0.95 (0.86-1.03) | 0.218 |
|  | XIV Diseases of the genitourinary system | 802/2086 | 0.93 (0.85-1.02) | 0.122 |
|  | CKD | 14/51 | 0.69 (0.41-1.16) | 0.163 |
|  | XVIII Symptoms, signs and abnormal clinical and laboratory findings, not elsewhere classified | 1213/3182 | 0.91 (0.83-1.00) | 0.043* |
|  | XIX Injury, poisoning and certain other consequences of external causes | 857/2273 | 0.94 (0.86-1.03) | 0.158 |
|  | XXI Factors influencing health status and contact with health services | 1646/4144 | 0.93 (0.82-1.05) | 0.259 |
| From mild AD dementia to institutionalization | I Certain infectious and parasitic diseases | 150/675 | 1.28 (1.06-1.53) | 0.009* |
|  | II Neoplasms | 258/1331 | 0.99 (0.85-1.16) | 0.938 |
|  | III Diseases of the blood and blood-forming organs and certain disorders involving the immune mechanism | 76/321 | 1.34 (1.06-1.71) | 0.016* |
|  | IV Endocrine, nutritional and metabolic diseases | 244/1168 | 1.18 (1.01-1.39) | 0.038* |
|  | T2DM | 95/368 | 1.51 (1.21-1.88) | 0.000* |
|  | V Mental, Behavioral and Neurodevelopmental disorders | 158/751 | 1.43 (1.19-1.72) | 0.000* |
|  | Depression | 76/398 | 1.11 (0.87-1.41) | 0.387 |
|  | VI Diseases of the nervous system | 198/967 | 1.31 (1.11-1.55) | 0.001* |
|  | VII Diseases of the eye and adnexa | 384/1873 | 1.08 (0.92-1.27) | 0.318 |
|  | VIII Diseases of the ear and mastoid process | 141/833 | 0.85 (0.71-1.03) | 0.097 |
|  | IX Diseases of the circulatory system | 428/2031 | 1.33 (1.13-1.57) | 0.001* |
|  | IHD | 113/525 | 1.13 (0.92-1.39) | 0.240 |
|  | Stroke | 72/250 | 1.62 (1.26-2.07) | 0.000* |
|  | X Diseases of the respiratory system | 154/824 | 1.04 (0.87-1.24) | 0.682 |
|  | XI Diseases of the digestive system | 304/1516 | 1.14 (0.98-1.34) | 0.087 |
|  | IBD | 10/50 | 1.12 (0.60-2.09) | 0.726 |
|  | XII Diseases of the skin and subcutaneous tissue | 208/1142 | 0.97 (0.82-1.15) | 0.732 |
|  | XIII Diseases of the musculoskeletal system and connective tissue | 356/1828 | 1.12 (0.96-1.31) | 0.152 |
|  | XIV Diseases of the genitourinary system | 302/1586 | 1.02 (0.88-1.19) | 0.772 |
|  | CKD | 14/51 | 1.58 (0.93-2.69) | 0.089 |
|  | XVIII Symptoms, signs and abnormal clinical and laboratory findings, not elsewhere classified | 473/2442 | 1.14 (0.96-1.35) | 0.134 |
|  | XIX Injury, poisoning and certain other consequences of external causes | 383/1799 | 1.41 (1.20-1.64) | <0.0001* |
|  | XXI Factors influencing health status and contact with health services | 570/3068 | 0.98 (0.78-1.24) | 0.880 |
| From mild AD dementia to death | I Certain infectious and parasitic diseases | 40/565 | 1.33 (0.93-1.89) | 0.120 |
|  | II Neoplasms | 75/1148 | 1.22 (0.90-1.65) | 0.207 |
|  | III Diseases of the blood and blood-forming organs and certain disorders involving the immune mechanism | 24/269 | 1.78 (1.16-2.75) | 0.009* |
|  | IV Endocrine, nutritional and metabolic diseases | 80/1004 | 1.85 (1.36-2.50) | <0.0001* |
|  | T2DM | 26/299 | 1.67 (1.10-2.54) | 0.017* |
|  | V Mental, Behavioral and Neurodevelopmental disorders | 56/649 | 2.45 (1.77-3.38) | <0.0001* |
|  | Depression | 30/352 | 2.03 (1.36-3.02) | 0.000* |
|  | VI Diseases of the nervous system | 63/832 | 1.57 (1.14-2.14) | 0.005* |
|  | VII Diseases of the eye and adnexa | 84/1573 | 0.79 (0.58-1.08) | 0.146 |
|  | VIII Diseases of the ear and mastoid process | 41/733 | 0.95 (0.67-1.35) | 0.771 |
|  | IX Diseases of the circulatory system | 126/1729 | 1.94 (1.36-2.76) | 0.000* |
|  | IHD | 44/456 | 1.56 (1.10-2.21) | 0.013* |
|  | Stroke | 23/201 | 1.95 (1.25-3.03) | 0.003* |
|  | X Diseases of the respiratory system | 54/724 | 1.54 (1.11-2.13) | 0.009* |
|  | XI Diseases of the digestive system | 88/1300 | 1.35 (1.00-1.83) | 0.054 |
|  | IBD | 1/41 | 0.42 (0.06-2.99) | 0.385 |
|  | XII Diseases of the skin and subcutaneous tissue | 46/980 | 0.80 (0.57-1.13) | 0.204 |
|  | XIII Diseases of the musculoskeletal system and connective tissue | 78/1550 | 0.88 (0.65-1.20) | 0.432 |
|  | XIV Diseases of the genitourinary system | 83/1367 | 1.21 (0.90-1.64) | 0.211 |
|  | CKD | 10/47 | 3.68 (1.93-7.00) | <0.0001* |
|  | XVIII Symptoms, signs and abnormal clinical and laboratory findings, not elsewhere classified | 129/2098 | 1.34 (0.93-1.92) | 0.112 |
|  | XIX Injury, poisoning and certain other consequences of external causes | 89/1505 | 1.23 (0.91-1.67) | 0.183 |
|  | XXI Factors influencing health status and contact with health services | 152/2650 | 1.43 (0.85-2.39) | 0.178 |
| From moderate to severe AD dementia | I Certain infectious and parasitic diseases | 124/577 | 0.98 (0.81-1.18) | 0.814 |
|  | II Neoplasms | 279/1203 | 0.93 (0.81-1.08) | 0.333 |
|  | III Diseases of the blood and blood-forming organs and certain disorders involving the immune mechanism | 48/276 | 0.77 (0.57-1.03) | 0.074 |
|  | IV Endocrine, nutritional and metabolic diseases | 219/1029 | 0.90 (0.77-1.05) | 0.195 |
|  | T2DM | 62/297 | 0.89 (0.68-1.15) | 0.363 |
|  | V Mental, Behavioral and Neurodevelopmental disorders | 170/718 | 0.86 (0.73-1.02) | 0.092 |
|  | Depression | 93/375 | 0.84 (0.67-1.04) | 0.104 |
|  | VI Diseases of the nervous system | 188/841 | 0.94 (0.80-1.11) | 0.450 |
|  | VII Diseases of the eye and adnexa | 373/1624 | 0.94 (0.81-1.08) | 0.370 |
|  | VIII Diseases of the ear and mastoid process | 169/700 | 0.93 (0.78-1.10) | 0.373 |
|  | IX Diseases of the circulatory system | 395/1772 | 0.91 (0.79-1.04) | 0.171 |
|  | IHD | 88/421 | 0.92 (0.73-1.15) | 0.467 |
|  | Stroke | 36/200 | 0.91 (0.65-1.28) | 0.590 |
|  | X Diseases of the respiratory system | 150/728 | 0.86 (0.72-1.02) | 0.090 |
|  | XI Diseases of the digestive system | 339/1388 | 1.00 (0.87-1.15) | 0.967 |
|  | IBD | 10/48 | 0.73 (0.39-1.36) | 0.321 |
|  | XII Diseases of the skin and subcutaneous tissue | 235/1013 | 0.98 (0.84-1.14) | 0.764 |
|  | XIII Diseases of the musculoskeletal system and connective tissue | 378/1630 | 0.92 (0.81-1.06) | 0.257 |
|  | XIV Diseases of the genitourinary system | 340/1438 | 0.96 (0.83-1.10) | 0.545 |
|  | CKD | 7/36 | 0.82 (0.39-1.74) | 0.609 |
|  | XVIII Symptoms, signs and abnormal clinical and laboratory findings, not elsewhere classified | 512/2206 | 0.92 (0.80-1.05) | 0.223 |
|  | XIX Injury, poisoning and certain other consequences of external causes | 370/1639 | 0.85 (0.74-0.97) | 0.019* |
|  | XXI Factors influencing health status and contact with health services | 698/2883 | 0.81 (0.67-0.96) | 0.017* |
| From moderate AD dementia to institutionalization | I Certain infectious and parasitic diseases | 302/755 | 1.21 (1.07-1.38) | 0.003* |
|  | II Neoplasms | 485/1409 | 0.97 (0.86-1.08) | 0.531 |
|  | III Diseases of the blood and blood-forming organs and certain disorders involving the immune mechanism | 130/358 | 1.08 (0.90-1.29) | 0.425 |
|  | IV Endocrine, nutritional and metabolic diseases | 477/1287 | 1.11 (0.99-1.24) | 0.073 |
|  | T2DM | 177/412 | 1.34 (1.14-1.57) | 0.000* |
|  | V Mental, Behavioral and Neurodevelopmental disorders | 357/905 | 1.33 (1.18-1.50) | <0.0001* |
|  | Depression | 194/476 | 1.23 (1.06-1.44) | 0.007* |
|  | VI Diseases of the nervous system | 371/1024 | 1.10 (0.97-1.23) | 0.136 |
|  | VII Diseases of the eye and adnexa | 717/1968 | 1.04 (0.94-1.16) | 0.428 |
|  | VIII Diseases of the ear and mastoid process | 286/817 | 0.95 (0.83-1.08) | 0.413 |
|  | IX Diseases of the circulatory system | 834/2211 | 1.12 (1.00-1.25) | 0.046* |
|  | IHD | 216/549 | 1.02 (0.88-1.18) | 0.800 |
|  | Stroke | 112/276 | 1.20 (0.98-1.45) | 0.071 |
|  | X Diseases of the respiratory system | 316/894 | 0.99 (0.87-1.13) | 0.896 |
|  | XI Diseases of the digestive system | 579/1628 | 0.96 (0.86-1.07) | 0.457 |
|  | IBD | 23/61 | 0.99 (0.66-1.50) | 0.977 |
|  | XII Diseases of the skin and subcutaneous tissue | 397/1175 | 0.95 (0.84-1.07) | 0.392 |
|  | XIII Diseases of the musculoskeletal system and connective tissue | 677/1929 | 0.97 (0.87-1.08) | 0.585 |
|  | XIV Diseases of the genitourinary system | 588/1686 | 1.01 (0.91-1.12) | 0.854 |
|  | CKD | 12/41 | 0.91 (0.52-1.61) | 0.756 |
|  | XVIII Symptoms, signs and abnormal clinical and laboratory findings, not elsewhere classified | 940/2634 | 1.01 (0.90-1.13) | 0.889 |
|  | XIX Injury, poisoning and certain other consequences of external causes | 682/1951 | 1.01 (0.90-1.12) | 0.916 |
|  | XXI Factors influencing health status and contact with health services | 1190/3375 | 1.01 (0.86-1.18) | 0.932 |
| From moderate AD dementia to death | I Certain infectious and parasitic diseases | 57/510 | 1.72 (1.26-2.36) | 0.001* |
|  | II Neoplasms | 102/1026 | 1.71 (1.28-2.27) | 0.000* |
|  | III Diseases of the blood and blood-forming organs and certain disorders involving the immune mechanism | 38/266 | 1.90 (1.32-2.72) | 0.000* |
|  | IV Endocrine, nutritional and metabolic diseases | 69/879 | 1.07 (0.80-1.44) | 0.656 |
|  | T2DM | 26/261 | 1.30 (0.86-1.98) | 0.213 |
|  | V Mental, Behavioral and Neurodevelopmental disorders | 40/588 | 1.16 (0.82-1.65) | 0.405 |
|  | Depression | 22/304 | 1.17 (0.75-1.83) | 0.499 |
|  | VI Diseases of the nervous system | 48/701 | 0.94 (0.68-1.30) | 0.701 |
|  | VII Diseases of the eye and adnexa | 117/1368 | 1.21 (0.90-1.63) | 0.209 |
|  | VIII Diseases of the ear and mastoid process | 54/585 | 1.26 (0.92-1.73) | 0.145 |
|  | IX Diseases of the circulatory system | 141/1518 | 1.67 (1.21-2.31) | 0.002* |
|  | IHD | 41/374 | 1.14 (0.80-1.62) | 0.461 |
|  | Stroke | 18/182 | 1.19 (0.73-1.94) | 0.491 |
|  | X Diseases of the respiratory system | 65/643 | 1.48 (1.09-2.00) | 0.011* |
|  | XI Diseases of the digestive system | 90/1139 | 0.99 (0.74-1.32) | 0.939 |
|  | IBD | 3/41 | 1.18 (0.38-3.70) | 0.775 |
|  | XII Diseases of the skin and subcutaneous tissue | 64/842 | 1.06 (0.78-1.43) | 0.703 |
|  | XIII Diseases of the musculoskeletal system and connective tissue | 103/1355 | 1.09 (0.82-1.45) | 0.567 |
|  | XIV Diseases of the genitourinary system | 86/1184 | 1.02 (0.77-1.36) | 0.890 |
|  | CKD | 8/37 | 2.42 (1.18-4.96) | 0.015* |
|  | XVIII Symptoms, signs and abnormal clinical and laboratory findings, not elsewhere classified | 134/1828 | 0.99 (0.73-1.36) | 0.968 |
|  | XIX Injury, poisoning and certain other consequences of external causes | 99/1368 | 1.05 (0.79-1.39) | 0.747 |
|  | XXI Factors influencing health status and contact with health services | 175/2360 | 1.48 (0.90-2.44) | 0.124 |
| From severe AD dementia to institutionalization | I Certain infectious and parasitic diseases | 121/173 | 0.97 (0.80-1.18) | 0.757 |
|  | II Neoplasms | 240/338 | 0.96 (0.82-1.12) | 0.619 |
|  | III Diseases of the blood and blood-forming organs and certain disorders involving the immune mechanism | 43/58 | 1.14 (0.83-1.55) | 0.414 |
|  | IV Endocrine, nutritional and metabolic diseases | 200/282 | 1.00 (0.85-1.17) | 0.953 |
|  | T2DM | 67/91 | 0.98 (0.76-1.26) | 0.872 |
|  | V Mental, Behavioral and Neurodevelopmental disorders | 175/249 | 0.97 (0.82-1.15) | 0.695 |
|  | Depression | 95/139 | 0.83 (0.67-1.03) | 0.099 |
|  | VI Diseases of the nervous system | 185/254 | 1.07 (0.90-1.26) | 0.443 |
|  | VII Diseases of the eye and adnexa | 330/461 | 1.14 (0.98-1.33) | 0.080 |
|  | VIII Diseases of the ear and mastoid process | 147/218 | 0.79 (0.65-0.95) | 0.011* |
|  | IX Diseases of the circulatory system | 363/498 | 1.06 (0.91-1.23) | 0.444 |
|  | IHD | 75/103 | 0.89 (0.69-1.13) | 0.330 |
|  | Stroke | 43/53 | 0.97 (0.71-1.33) | 0.857 |
|  | X Diseases of the respiratory system | 138/199 | 0.92 (0.76-1.11) | 0.369 |
|  | XI Diseases of the digestive system | 314/436 | 1.01 (0.87-1.17) | 0.861 |
|  | IBD | 8/14 | 0.75 (0.37-1.51) | 0.419 |
|  | XII Diseases of the skin and subcutaneous tissue | 193/285 | 1.03 (0.87-1.21) | 0.754 |
|  | XIII Diseases of the musculoskeletal system and connective tissue | 334/466 | 1.06 (0.91-1.23) | 0.447 |
|  | XIV Diseases of the genitourinary system | 297/438 | 0.85 (0.73-0.99) | 0.034* |
|  | CKD | 10/16 | 1.38 (0.74-2.59) | 0.311 |
|  | XVIII Symptoms, signs and abnormal clinical and laboratory findings, not elsewhere classified | 467/641 | 1.05 (0.90-1.22) | 0.530 |
|  | XIX Injury, poisoning and certain other consequences of external causes | 341/490 | 0.98 (0.84-1.13) | 0.741 |
|  | XXI Factors influencing health status and contact with health services | 615/848 | 0.94 (0.78-1.14) | 0.546 |
| From severe AD dementia to death | I Certain infectious and parasitic diseases | 27/79 | 1.01 (0.66-1.55) | 0.963 |
|  | II Neoplasms | 61/159 | 1.21 (0.87-1.70) | 0.254 |
|  | III Diseases of the blood and blood-forming organs and certain disorders involving the immune mechanism | 18/33 | 1.79 (1.08-2.98) | 0.025* |
|  | IV Endocrine, nutritional and metabolic diseases | 50/132 | 1.45 (1.02-2.04) | 0.036* |
|  | T2DM | 18/42 | 1.45 (0.88-2.38) | 0.146 |
|  | V Mental, Behavioral and Neurodevelopmental disorders | 35/109 | 0.91 (0.62-1.34) | 0.649 |
|  | Depression | 20/64 | 0.70 (0.43-1.12) | 0.138 |
|  | VI Diseases of the nervous system | 42/111 | 0.95 (0.66-1.37) | 0.783 |
|  | VII Diseases of the eye and adnexa | 76/207 | 1.11 (0.78-1.57) | 0.555 |
|  | VIII Diseases of the ear and mastoid process | 33/104 | 0.89 (0.60-1.32) | 0.575 |
|  | IX Diseases of the circulatory system | 84/219 | 1.16 (0.82-1.62) | 0.405 |
|  | IHD | 23/51 | 1.19 (0.75-1.88) | 0.465 |
|  | Stroke | 14/24 | 1.34 (0.77-2.33) | 0.308 |
|  | X Diseases of the respiratory system | 43/104 | 1.29 (0.90-1.85) | 0.165 |
|  | XI Diseases of the digestive system | 76/198 | 1.08 (0.77-1.51) | 0.647 |
|  | IBD | 4/10 | 1.34 (0.49-3.65) | 0.567 |
|  | XII Diseases of the skin and subcutaneous tissue | 45/137 | 1.18 (0.82-1.69) | 0.381 |
|  | XIII Diseases of the musculoskeletal system and connective tissue | 68/200 | 0.92 (0.66-1.29) | 0.636 |
|  | XIV Diseases of the genitourinary system | 70/211 | 0.93 (0.67-1.30) | 0.673 |
|  | CKD | 1/7 | 0.60 (0.08-4.33) | 0.612 |
|  | XVIII Symptoms, signs and abnormal clinical and laboratory findings, not elsewhere classified | 110/284 | 1.37 (0.93-2.03) | 0.113 |
|  | XIX Injury, poisoning and certain other consequences of external causes | 84/233 | 1.21 (0.87-1.70) | 0.264 |
|  | XXI Factors influencing health status and contact with health services | 124/357 | 0.94 (0.58-1.51) | 0.792 |
| From institutionalized very mild AD dementia to institutionalized mild AD dementia | I Certain infectious and parasitic diseases | 11/55 | 1.15 (0.55-2.41) | 0.714 |
|  | II Neoplasms | 12/92 | 0.48 (0.23-0.99) | 0.048* |
|  | III Diseases of the blood and blood-forming organs and certain disorders involving the immune mechanism | 3/28 | 0.74 (0.22-2.46) | 0.618 |
|  | IV Endocrine, nutritional and metabolic diseases | 17/87 | 1.26 (0.64-2.48) | 0.506 |
|  | T2DM | 4/31 | 0.94 (0.32-2.76) | 0.911 |
|  | V Mental, Behavioral and Neurodevelopmental disorders | 13/83 | 0.67 (0.30-1.50) | 0.326 |
|  | Depression | 10/48 | 1.29 (0.59-2.81) | 0.520 |
|  | VI Diseases of the nervous system | 11/73 | 0.95 (0.46-1.97) | 0.896 |
|  | VII Diseases of the eye and adnexa | 18/119 | 0.69 (0.34-1.42) | 0.315 |
|  | VIII Diseases of the ear and mastoid process | 4/49 | 0.63 (0.21-1.89) | 0.411 |
|  | IX Diseases of the circulatory system | 26/148 | 0.98 (0.44-2.21) | 0.967 |
|  | IHD | 11/35 | 2.21 (1.04-4.65) | 0.038* |
|  | Stroke | 1/28 | 0.21 (0.03-1.53) | 0.123 |
|  | X Diseases of the respiratory system | 11/53 | 1.04 (0.49-2.22) | 0.919 |
|  | XI Diseases of the digestive system | 14/90 | 1.15 (0.55-2.40) | 0.714 |
|  | IBD | 0/3 | - | - |
|  | XII Diseases of the skin and subcutaneous tissue | 11/82 | 0.83 (0.40-1.72) | 0.619 |
|  | XIII Diseases of the musculoskeletal system and connective tissue | 21/119 | 1.06 (0.52-2.16) | 0.880 |
|  | XIV Diseases of the genitourinary system | 17/111 | 0.75 (0.37-1.49) | 0.406 |
|  | CKD | 0/3 | - | - |
|  | XVIII Symptoms, signs and abnormal clinical and laboratory findings, not elsewhere classified | 27/164 | 0.68 (0.30-1.55) | 0.363 |
|  | XIX Injury, poisoning and certain other consequences of external causes | 27/139 | 2.11 (0.94-4.72) | 0.070 |
|  | XXI Factors influencing health status and contact with health services | 34/187 | 4.78 (0.64-35.79) | 0.128 |
| From institutionalized very mild AD dementia to death | I Certain infectious and parasitic diseases | 7/51 | 1.57 (0.58-4.21) | 0.372 |
|  | II Neoplasms | 9/89 | 1.09 (0.41-2.92) | 0.861 |
|  | III Diseases of the blood and blood-forming organs and certain disorders involving the immune mechanism | 8/33 | 4.68 (1.68-13.08) | 0.003* |
|  | IV Endocrine, nutritional and metabolic diseases | 7/77 | 0.95 (0.36-2.51) | 0.919 |
|  | T2DM | 3/30 | 1.15 (0.32-4.09) | 0.828 |
|  | V Mental, Behavioral and Neurodevelopmental disorders | 6/76 | 0.85 (0.31-2.38) | 0.761 |
|  | Depression | 3/41 | 1.02 (0.28-3.64) | 0.977 |
|  | VI Diseases of the nervous system | 5/67 | 0.72 (0.25-2.08) | 0.549 |
|  | VII Diseases of the eye and adnexa | 9/110 | 0.71 (0.26-1.95) | 0.505 |
|  | VIII Diseases of the ear and mastoid process | 5/50 | 1.51 (0.49-4.67) | 0.471 |
|  | IX Diseases of the circulatory system | 11/133 | 0.59 (0.21-1.63) | 0.306 |
|  | IHD | 3/27 | 0.99 (0.26-3.75) | 0.991 |
|  | Stroke | 3/30 | 1.01 (0.29-3.54) | 0.984 |
|  | X Diseases of the respiratory system | 7/49 | 1.93 (0.73-5.15) | 0.188 |
|  | XI Diseases of the digestive system | 6/82 | 0.59 (0.21-1.63) | 0.311 |
|  | IBD | 0/3 | - | - |
|  | XII Diseases of the skin and subcutaneous tissue | 3/74 | 0.32 (0.09-1.13) | 0.076 |
|  | XIII Diseases of the musculoskeletal system and connective tissue | 9/107 | 0.77 (0.28-2.06) | 0.597 |
|  | XIV Diseases of the genitourinary system | 8/102 | 0.76 (0.28-2.06) | 0.594 |
|  | CKD | 1/4 | 2.48 (0.32-19.22) | 0.384 |
|  | XVIII Symptoms, signs and abnormal clinical and laboratory findings, not elsewhere classified | 16/153 | 3.85 (0.51-29.17) | 0.193 |
|  | XIX Injury, poisoning and certain other consequences of external causes | 15/127 | 3.68 (0.84-16.14) | 0.085 |
|  | XXI Factors influencing health status and contact with health services | 17/170 | - | - |
| From institutionalized mild AD dementia to institutionalized moderate AD dementia | I Certain infectious and parasitic diseases | 23/190 | 0.85 (0.53-1.38) | 0.515 |
|  | II Neoplasms | 40/269 | 1.13 (0.75-1.72) | 0.553 |
|  | III Diseases of the blood and blood-forming organs and certain disorders involving the immune mechanism | 11/103 | 0.80 (0.43-1.52) | 0.502 |
|  | IV Endocrine, nutritional and metabolic diseases | 32/276 | 0.78 (0.51-1.21) | 0.275 |
|  | T2DM | 14/107 | 1.11 (0.62-1.98) | 0.732 |
|  | V Mental, Behavioral and Neurodevelopmental disorders | 25/210 | 0.70 (0.44-1.12) | 0.136 |
|  | Depression | 19/110 | 0.97 (0.57-1.64) | 0.907 |
|  | VI Diseases of the nervous system | 27/232 | 0.86 (0.54-1.37) | 0.532 |
|  | VII Diseases of the eye and adnexa | 54/412 | 0.90 (0.59-1.38) | 0.642 |
|  | VIII Diseases of the ear and mastoid process | 18/152 | 0.74 (0.44-1.26) | 0.271 |
|  | IX Diseases of the circulatory system | 60/486 | 0.62 (0.40-0.96) | 0.031* |
|  | IHD | 13/120 | 0.93 (0.51-1.70) | 0.824 |
|  | Stroke | 8/87 | 0.74 (0.36-1.54) | 0.419 |
|  | X Diseases of the respiratory system | 22/175 | 1.00 (0.62-1.62) | 0.994 |
|  | XI Diseases of the digestive system | 41/330 | 0.92 (0.61-1.39) | 0.690 |
|  | IBD | 1/12 | 0.54 (0.08-3.90) | 0.543 |
|  | XII Diseases of the skin and subcutaneous tissue | 27/232 | 0.79 (0.50-1.24) | 0.304 |
|  | XIII Diseases of the musculoskeletal system and connective tissue | 57/395 | 1.33 (0.88-2.03) | 0.180 |
|  | XIV Diseases of the genitourinary system | 38/346 | 0.76 (0.50-1.15) | 0.197 |
|  | CKD | 1/18 | 0.74 (0.10-5.47) | 0.767 |
|  | XVIII Symptoms, signs and abnormal clinical and laboratory findings, not elsewhere classified | 67/518 | 0.73 (0.46-1.16) | 0.186 |
|  | XIX Injury, poisoning and certain other consequences of external causes | 57/446 | 0.72 (0.47-1.10) | 0.125 |
|  | XXI Factors influencing health status and contact with health services | 83/602 | 0.89 (0.46-1.73) | 0.726 |
| From institutionalized mild AD dementia to death | I Certain infectious and parasitic diseases | 21/188 | 0.98 (0.58-1.66) | 0.934 |
|  | II Neoplasms | 37/266 | 1.83 (1.12-2.99) | 0.015* |
|  | III Diseases of the blood and blood-forming organs and certain disorders involving the immune mechanism | 9/101 | 0.82 (0.40-1.67) | 0.584 |
|  | IV Endocrine, nutritional and metabolic diseases | 34/278 | 1.48 (0.91-2.39) | 0.114 |
|  | T2DM | 11/104 | 0.97 (0.50-1.88) | 0.919 |
|  | V Mental, Behavioral and Neurodevelopmental disorders | 19/204 | 0.78 (0.45-1.34) | 0.373 |
|  | Depression | 14/105 | 1.20 (0.66-2.20) | 0.544 |
|  | VI Diseases of the nervous system | 26/231 | 1.11 (0.67-1.84) | 0.688 |
|  | VII Diseases of the eye and adnexa | 43/401 | 1.11 (0.67-1.83) | 0.683 |
|  | VIII Diseases of the ear and mastoid process | 17/151 | 1.09 (0.62-1.89) | 0.773 |
|  | IX Diseases of the circulatory system | 54/480 | 1.26 (0.69-2.30) | 0.445 |
|  | IHD | 27/134 | 2.28 (1.38-3.74) | 0.001* |
|  | Stroke | 13/92 | 1.33 (0.72-2.48) | 0.366 |
|  | X Diseases of the respiratory system | 31/184 | 2.19 (1.35-3.55) | 0.002* |
|  | XI Diseases of the digestive system | 34/323 | 1.10 (0.68-1.77) | 0.706 |
|  | CKD | 3/20 | 1.90 (0.58-6.20) | 0.285 |
|  | XII Diseases of the skin and subcutaneous tissue | 17/222 | 0.61 (0.35-1.08) | 0.088 |
|  | XIII Diseases of the musculoskeletal system and connective tissue | 35/373 | 0.83 (0.51-1.35) | 0.461 |
|  | XIV Diseases of the genitourinary system | 37/345 | 1.14 (0.70-1.85) | 0.588 |
|  | IBD | 1/12 | 0.65 (0.09-4.70) | 0.670 |
|  | XVIII Symptoms, signs and abnormal clinical and laboratory findings, not elsewhere classified | 58/509 | 1.50 (0.76-2.96) | 0.239 |
|  | XIX Injury, poisoning and certain other consequences of external causes | 46/435 | 1.08 (0.64-1.80) | 0.775 |
|  | XXI Factors influencing health status and contact with health services | 58/577 | 0.72 (0.37-1.42) | 0.344 |
| From institutionalized moderate AD dementia to institutionalized severe AD dementia | I Certain infectious and parasitic diseases | 67/377 | 1.00 (0.76-1.33) | 0.981 |
|  | II Neoplasms | 101/522 | 0.92 (0.71-1.19) | 0.503 |
|  | III Diseases of the blood and blood-forming organs and certain disorders involving the immune mechanism | 21/162 | 0.74 (0.47-1.16) | 0.187 |
|  | IV Endocrine, nutritional and metabolic diseases | 83/548 | 0.78 (0.60-1.02) | 0.073 |
|  | T2DM | 31/196 | 0.90 (0.62-1.32) | 0.597 |
|  | V Mental, Behavioral and Neurodevelopmental disorders | 65/414 | 0.87 (0.65-1.15) | 0.326 |
|  | Depression | 39/230 | 0.84 (0.60-1.19) | 0.335 |
|  | VI Diseases of the nervous system | 89/424 | 1.11 (0.85-1.44) | 0.438 |
|  | VII Diseases of the eye and adnexa | 137/777 | 1.08 (0.83-1.40) | 0.564 |
|  | VIII Diseases of the ear and mastoid process | 45/315 | 0.70 (0.50-0.97) | 0.031* |
|  | IX Diseases of the circulatory system | 170/922 | 1.04 (0.79-1.37) | 0.770 |
|  | IHD | 42/226 | 1.12 (0.80-1.58) | 0.508 |
|  | Stroke | 23/147 | 0.94 (0.61-1.44) | 0.765 |
|  | X Diseases of the respiratory system | 61/374 | 0.85 (0.63-1.14) | 0.275 |
|  | XI Diseases of the digestive system | 112/643 | 1.01 (0.78-1.29) | 0.969 |
|  | IBD | 3/28 | 0.97 (0.31-3.03) | 0.956 |
|  | XII Diseases of the skin and subcutaneous tissue | 78/435 | 1.02 (0.78-1.35) | 0.868 |
|  | XIII Diseases of the musculoskeletal system and connective tissue | 132/751 | 0.90 (0.70-1.16) | 0.427 |
|  | XIV Diseases of the genitourinary system | 122/678 | 1.03 (0.81-1.33) | 0.792 |
|  | CKD | 4/16 | 1.76 (0.64-4.79) | 0.272 |
|  | XVIII Symptoms, signs and abnormal clinical and laboratory findings, not elsewhere classified | 180/1045 | 0.90 (0.68-1.18) | 0.431 |
|  | XIX Injury, poisoning and certain other consequences of external causes | 148/828 | 1.03 (0.80-1.33) | 0.817 |
|  | XXI Factors influencing health status and contact with health services | 232/1264 | 1.14 (0.75-1.74) | 0.548 |
| From institutionalized moderate AD dementia to death | I Certain infectious and parasitic diseases | 51/361 | 1.30 (0.92-1.84) | 0.135 |
|  | II Neoplasms | 70/491 | 1.48 (1.07-2.04) | 0.019* |
|  | III Diseases of the blood and blood-forming organs and certain disorders involving the immune mechanism | 31/172 | 1.43 (0.95-2.15) | 0.084 |
|  | IV Endocrine, nutritional and metabolic diseases | 67/532 | 1.11 (0.80-1.53) | 0.541 |
|  | T2DM | 29/194 | 1.10 (0.73-1.67) | 0.650 |
|  | V Mental, Behavioral and Neurodevelopmental disorders | 47/396 | 1.17 (0.82-1.67) | 0.372 |
|  | Depression | 27/218 | 1.14 (0.75-1.75) | 0.534 |
|  | VI Diseases of the nervous system | 61/396 | 1.47 (1.06-2.04) | 0.023* |
|  | VII Diseases of the eye and adnexa | 89/729 | 1.02 (0.73-1.42) | 0.925 |
|  | VIII Diseases of the ear and mastoid process | 29/299 | 0.79 (0.53-1.19) | 0.266 |
|  | IX Diseases of the circulatory system | 112/864 | 1.28 (0.88-1.85) | 0.199 |
|  | IHD | 32/216 | 1.19 (0.80-1.77) | 0.398 |
|  | Stroke | 19/143 | 0.95 (0.58-1.55) | 0.823 |
|  | X Diseases of the respiratory system | 53/366 | 1.25 (0.89-1.76) | 0.198 |
|  | XI Diseases of the digestive system | 73/604 | 1.09 (0.78-1.51) | 0.620 |
|  | IBD | 1/26 | 0.67 (0.09-4.82) | 0.692 |
|  | XII Diseases of the skin and subcutaneous tissue | 49/406 | 1.04 (0.74-1.47) | 0.816 |
|  | XIII Diseases of the musculoskeletal system and connective tissue | 86/705 | 1.08 (0.78-1.51) | 0.635 |
|  | XIV Diseases of the genitourinary system | 82/638 | 1.37 (0.99-1.90) | 0.056 |
|  | CKD | 7/19 | 3.51 (1.57-7.85) | 0.002* |
|  | XVIII Symptoms, signs and abnormal clinical and laboratory findings, not elsewhere classified | 119/984 | 1.21 (0.82-1.80) | 0.342 |
|  | XIX Injury, poisoning and certain other consequences of external causes | 92/772 | 1.05 (0.76-1.46) | 0.767 |
|  | XXI Factors influencing health status and contact with health services | 133/1165 | 1.10 (0.67-1.81) | 0.712 |
| From institutionalized severe AD dementia to death | I Certain infectious and parasitic diseases | 167/243 | 1.10 (0.92-1.32) | 0.294 |
|  | II Neoplasms | 245/381 | 1.11 (0.95-1.30) | 0.195 |
|  | III Diseases of the blood and blood-forming organs and certain disorders involving the immune mechanism | 62/87 | 1.32 (1.02-1.73) | 0.037* |
|  | IV Endocrine, nutritional and metabolic diseases | 234/337 | 1.22 (1.04-1.44) | 0.014* |
|  | T2DM | 85/117 | 1.34 (1.06-1.68) | 0.014* |
|  | V Mental, Behavioral and Neurodevelopmental disorders | 164/280 | 1.00 (0.84-1.19) | 0.984 |
|  | Depression | 89/152 | 0.92 (0.74-1.15) | 0.466 |
|  | VI Diseases of the nervous system | 216/319 | 1.11 (0.94-1.31) | 0.216 |
|  | VII Diseases of the eye and adnexa | 331/525 | 1.11 (0.95-1.29) | 0.199 |
|  | VIII Diseases of the ear and mastoid process | 129/207 | 0.92 (0.76-1.12) | 0.432 |
|  | IX Diseases of the circulatory system | 423/618 | 1.28 (1.09-1.51) | 0.003* |
|  | IHD | 100/134 | 1.19 (0.95-1.48) | 0.127 |
|  | Stroke | 57/88 | 0.93 (0.71-1.23) | 0.624 |
|  | X Diseases of the respiratory system | 166/246 | 1.17 (0.98-1.40) | 0.089 |
|  | XI Diseases of the digestive system | 310/477 | 1.10 (0.94-1.28) | 0.242 |
|  | IBD | 7/12 | 1.45 (0.68-3.05) | 0.334 |
|  | XII Diseases of the skin and subcutaneous tissue | 195/307 | 1.08 (0.91-1.27) | 0.388 |
|  | XIII Diseases of the musculoskeletal system and connective tissue | 329/508 | 1.09 (0.94-1.28) | 0.254 |
|  | XIV Diseases of the genitourinary system | 307/489 | 1.00 (0.85-1.16) | 0.964 |
|  | CKD | 12/17 | 1.70 (0.95-3.03) | 0.073 |
|  | XVIII Symptoms, signs and abnormal clinical and laboratory findings, not elsewhere classified | 477/749 | 1.14 (0.96-1.35) | 0.134 |
|  | XIX Injury, poisoning and certain other consequences of external causes | 386/593 | 1.13 (0.96-1.32) | 0.136 |
|  | XXI Factors influencing health status and contact with health services | 592/916 | 1.17 (0.92-1.49) | 0.201 |

Abbreviations: AD = Alzheimer’s disease; CI = confidence interval; CKD = chronic kidney disease; HR = hazard ratio; IBD = inflammatory bowel disease; ICD-10 = the 10th revision of the International Classification of Diseases; IHD = ischemic heart disease; T2DM = type 2 diabetes.

Notes: ICD codes related to dementia (F00, F01, F02, F03, F05, F06, G30, G31, and R41) were excluded from chapters V, VI, and XVIII.

The following ICD chapters were excluded due to lack of relevance and few numbers of people with these conditions in this study: XV (Pregnancy, childbirth and the puerperium), XVI (Certain conditions originating in the perinatal period), and XX (External causes of morbidity).

# Table S5. Hazard ratios for significant ICD-10 chapters simultaneously included in one multistate model

| Transition | Predictor | No. events/No. people at risk | | HR (95% CI) | | P-value |
| --- | --- | --- | --- | --- | --- | --- |
| From very mild to mild AD dementia | VII Diseases of the eye and adnexa | 539/1263 | | 0.89 (0.79-1.00) | | 0.046* |
|  | X Diseases of the respiratory system | 223/550 | | 0.87 (0.75-1.01) | | 0.068 |
|  | XXI Factors influencing health status and contact with health services | 1009/2223 | | 0.89 (0.75-1.05) | | 0.156 |
| From very mild AD dementia to institutionalization | V Mental, Behavioral and Neurodevelopmental disorders | 60/333 | | 1.86 (1.37-2.52) | | <0.0001* |
|  | XIX Injury, poisoning and certain other consequences of external causes | 125/826 | | 1.70 (1.28-2.28) | | 0.000* |
| From very mild AD dementia to death | I Certain infectious and parasitic diseases | 15/262 | | 1.63 (0.87-3.06) | | 0.129 |
|  | IX Diseases of the circulatory system | 37/775 | | 1.82 (0.92-3.58) | | 0.085 |
|  | X Diseases of the respiratory system | 19/346 | | 1.58 (0.88-2.86) | | 0.128 |
| From mild to moderate AD dementia | XVIII Symptoms, signs and abnormal clinical and laboratory findings, not elsewhere classified | 1213/3182 | | 0.90 (0.83-1.00) | | 0.043* |
| From mild AD dementia to institutionalization | I Certain infectious and parasitic diseases | 150/675 | | 1.15 (0.96-1.39) | | 0.140 |
|  | III Diseases of the blood and blood-forming organs and certain disorders involving the immune mechanism | 76/321 | | 1.16 (0.91-1.49) | | 0.223 |
|  | IV Endocrine, nutritional and metabolic diseases | 244/1168 | | 1.02 (0.86-1.21) | | 0.826 |
|  | V Mental, Behavioral and Neurodevelopmental disorders | 158/751 | | 1.32 (1.10-1.59) | | 0.003* |
|  | VI Diseases of the nervous system | 198/967 | | 1.17 (0.99-1.40) | | 0.066 |
|  | IX Diseases of the circulatory system | 428/2031 | | 1.19 (0.99-1.41) | | 0.059 |
|  | XIX Injury, poisoning and certain other consequences of external causes | 383/1799 | | 1.31 (1.11-1.53) | | 0.001* |
| From mild AD dementia to death | III Diseases of the blood and blood-forming organs and certain disorders involving the immune mechanism | 24/269 | | 1.38 (0.88-2.14) | | 0.157 |
|  | IV Endocrine, nutritional and metabolic diseases | 80/1004 | | 1.40 (1.01-1.94) | | 0.042* |
|  | V Mental, Behavioral and Neurodevelopmental disorders | 56/649 | | 2.05 (1.47-2.87) | | <0.0001* |
|  | VI Diseases of the nervous system | 63/832 | | 1.27 (0.92-1.76) | | 0.138 |
|  | IX Diseases of the circulatory system | 126/1729 | | 1.46 (1.00-2.15) | | 0.049* |
|  | X Diseases of the respiratory system | 54/724 | | 1.20 (0.86-1.68) | | 0.290 |
| From moderate to severe AD dementia | XIX Injury, poisoning and certain other consequences of external causes | 370/1639 | | 0.87 (0.76-1.00) | | 0.045* |
|  | XXI Factors influencing health status and contact with health services | 698/2883 | | 0.83 (0.69-0.99) | | 0.042* |
| From moderate AD dementia to institutionalization | I Certain infectious and parasitic diseases | 302/755 | | 1.17 (1.03-1.34) | | 0.016* |
|  | V Mental, Behavioral and Neurodevelopmental disorders | 357/905 | | 1.30 (1.15-1.47) | | <0.0001* |
|  | IX Diseases of the circulatory system | 834/2211 | | 1.08 (0.97-1.21) | | 0.169 |
| From moderate AD dementia to death | I Certain infectious and parasitic diseases | 57/510 | | 1.38 (0.99-1.92) | | 0.055 |
|  | II Neoplasms | 102/1026 | | 1.57 (1.18-2.09) | | 0.002* |
|  | III Diseases of the blood and blood-forming organs and certain disorders involving the immune mechanism | 38/266 | | 1.58 (1.10-2.30) | | 0.015* |
|  | IX Diseases of the circulatory system | 141/1518 | | 1.45 (1.04-2.03) | | 0.029* |
|  | X Diseases of the respiratory system | 65/643 | | 1.31 (0.96-1.78) | | 0.085 |
| From severe AD dementia to institutionalization | VIII Diseases of the ear and mastoid process | 147/218 | | 0.80 (0.67-0.97) | | 0.020* |
|  | XIV Diseases of the genitourinary system | 297/438 | | 0.87 (0.75-1.01) | | 0.068 |
| From severe AD dementia to death | III Diseases of the blood and blood-forming organs and certain disorders involving the immune mechanism | 18/33 | | 1.51 (0.86-2.67) | | 0.153 |
|  | IV Endocrine, nutritional and metabolic diseases | 50/132 | | 1.30 (0.88-1.90) | | 0.184 |
| From institutionalized very mild AD dementia to institutionalized mild AD dementia | II Neoplasms | 12/92 | | 0.48 (0.23-0.99) | | 0.048* |
| From institutionalized very mild AD dementia to death | III Diseases of the blood and blood-forming organs and certain disorders involving the immune mechanism | 8/33 | | 4.66 (1.68-13.08) | | 0.003* |
| From institutionalized mild AD dementia to institutionalized moderate AD dementia | IX Diseases of the circulatory system | 60/486 | | 0.61 (0.40-0.96) | | 0.031* |
| From institutionalized mild AD dementia to death | II Neoplasms | 37/266 | | 1.73 (1.06-2.83) | | 0.028* |
|  | X Diseases of the respiratory system | 31/184 | | 2.10 (1.29-3.40) | | 0.003* |
| From institutionalized moderate AD dementia to institutionalized severe AD dementia | VIII Diseases of the ear and mastoid process | 45/315 | | 0.70 (0.50-0.97) | | 0.031* |
| From institutionalized moderate AD dementia to death | II Neoplasms | 70/491 | | 1.45 (1.05-2.01) | | 0.024* |
|  | VI Diseases of the nervous system | 61/396 | | 1.45 (1.04-2.01) | | 0.029* |
| From institutionalized severe AD dementia to death | III Diseases of the blood and blood-forming organs and certain disorders involving the immune mechanism | 62/87 | | 1.25 (0.95-1.63) | | 0.108 |
|  | IV Endocrine, nutritional and metabolic diseases | 234/337 | 1.14 (0.96-1.35) | | 0.124 | |
|  | IX Diseases of the circulatory system | 423/618 | 1.21 (1.02-1.44) | | 0.027* | |

Abbreviations: AD = Alzheimer’s disease; CI = confidence interval; HR = hazard ratio, ICD-10 = the 10th revision of the International Classification of Diseases.

Notes: ICD codes related to dementia (F00, F01, F02, F03, F05, F06, G30, G31, and R41) were excluded from chapters V, VI, and XVIII.

The results were derived from age- and sex-adjusted multistate models, incorporating all comorbidities associated with AD dementia stages as listed in Table S4.

*P-value <0.05.

# Table S6. Interactions between comorbidity groups associated with AD dementia prognosis and age and sex

| Transition | Predictor | HR (95% CI) for predictor | HR (95% CI) for interaction with age | HR (95% CI) for interaction with sex |
| --- | --- | --- | --- | --- |
| From very mild to mild AD dementia | VII Diseases of the eye and adnexa | 0.82 (0.68-0.97) | 0.99 (0.97-1.00) | 1.12 (0.89-1.41) |
| From very mild AD dementia to institutionalization | V Mental, Behavioral and Neurodevelopmental disorders | 2.48 (1.56-3.94) | 0.98 (0.94-1.02) | 0.64 (0.35-1.19) |
|  | XIX Injury, poisoning and certain other consequences of external causes | 2.12 (1.31-3.40) | 0.97 (0.93-1.02) | 0.79 (0.44-1.42) |
| From mild to moderate AD dementia | XVIII Symptoms, signs and abnormal clinical and laboratory findings, not elsewhere classified | 0.93 (0.81-1.08) | 1.00 (0.99-1.02) | 0.96 (0.80-1.16) |
| From mild AD dementia to institutionalization | V Mental, Behavioral and Neurodevelopmental disorders | 1.67 (1.25-2.23) | 1.01 (0.98-1.04) | 0.71 (0.49-1.03)** |
|  | XIX Injury, poisoning and certain other consequences of external causes | 1.43 (1.11-1.84) | 1.00 (0.98-1.03) | 0.92 (0.67-1.27) |
| From mild AD dementia to death | IV Endocrine, nutritional and metabolic diseases | 1.26 (0.78-2.02) | 0.99 (0.94-1.04) | 1.73 (0.88-3.37) |
|  | V Mental, Behavioral and Neurodevelopmental disorders | 2.80 (1.80-4.36) | 0.99 (0.94-1.04) | 0.57 (0.29-1.12) |
|  | IX Diseases of the circulatory system | 1.15 (0.70-1.89) | 1.05 (0.99-1.11)** | 1.62 (0.75-3.51) |
| From moderate to severe AD dementia | XIX Injury, poisoning and certain other consequences of external causes | 0.84 (0.68-1.04) | 1.00 (0.98-1.01) | 1.05 (0.79-1.38) |
|  | XXI Factors influencing health status and contact with health services | 0.84 (0.64-1.10) | 1.00 (0.98-1.02) | 0.97 (0.68-1.39) |
| From moderate AD dementia to institutionalization | I Certain infectious and parasitic diseases | 1.12 (0.90-1.37) | 1.01 (0.99-1.03) | 1.07 (0.82-1.39) |
|  | V Mental, Behavioral and Neurodevelopmental disorders | 1.42 (1.17-1.72) | 0.98 (0.97-1.00)** | 0.90 (0.70-1.15) |
| From moderate AD dementia to death | II Neoplasms | 1.68 (1.07-2.63) | 1.00 (0.96-1.05) | 0.90 (0.50-1.63) |
|  | III Diseases of the blood and blood-forming organs and certain disorders involving the immune mechanism | 1.13 (0.57-2.23) | 1.04 (0.98-1.11) | 1.34 (0.63-2.81) |
|  | IX Diseases of the circulatory system | 1.60 (0.97-2.64) | 1.02 (0.97-1.07) | 0.77 (0.39-1.51) |
| From severe AD dementia to institutionalization | VIII Diseases of the ear and mastoid process | 0.77 (0.58-1.03) | 1.00 (0.98-1.02) | 1.02 (0.71-1.48) |
| From institutionalized very mild AD dementia to institutionalized mild AD dementia | II Neoplasms | - | - | - |
| From institutionalized very mild AD dementia to death | III Diseases of the blood and blood-forming organs and certain disorders involving the immune mechanism | - | - | - |
| From institutionalized mild AD dementia to institutionalized moderate AD dementia | IX Diseases of the circulatory system | 0.51 (0.26-1.02) | 1.03 (0.97-1.09) | 1.42 (0.59-3.43) |
| From institutionalized mild AD dementia to death | II Neoplasms | 1.25 (0.65-2.36) | 1.00 (0.93-1.08) | 2.25 (0.83-6.04) |
|  | X Diseases of the respiratory system | 1.48 (0.76-2.87) | 1.00 (0.93-1.08) | 2.23 (0.82-5.98) |
| From institutionalized moderate AD dementia to institutionalized severe AD dementia | VIII Diseases of the ear and mastoid process | 0.66 (0.41-1.09) | 0.98 (0.93-1.03) | 1.08 (0.56-2.08) |
| From institutionalized moderate AD dementia to death | II Neoplasms | 1.70 (1.07-2.70) | 0.99 (0.94-1.04) | 0.76 (0.39-1.46) |
|  | VI Diseases of the nervous system | 1.70 (1.07-2.69) | 0.98 (0.93-1.03) | 0.78 (0.40-1.52) |
| From institutionalized severe AD dementia to death | IX Diseases of the circulatory system | 1.30 (1.01-1.65) | 0.99 (0.97-1.01) | 0.98 (0.71-1.35) |

Abbreviations: AD = Alzheimer’s disease; CI = confidence interval; HR = hazard ratio.

Notes: ICD codes related to dementia (F00, F01, F02, F03, F05, F06, G30, G31, and R41) were excluded from chapters V.

The results were derived from multistate models that included all comorbidities listed in the table as covariates. Transitions with <20 events were excluded from the analyses.

**P-value for interaction <0.1.

# Table S7. Comorbidity groups by human organ systems significantly associated with AD dementia prognosis, adjusted for CSF biomarkers

| Transition | Predictor | No. events/No. people at risk | HR (95% CI) | P-value |
| --- | --- | --- | --- | --- |
| From very mild to mild AD dementia | VII Diseases of the eye and adnexa | 539/1263 | 0.87 (0.77-0.98) | 0.019* |
| From very mild AD dementia to institutionalization | V Mental, Behavioral and Neurodevelopmental disorders | 60/333 | 1.86 (1.37-2.53) | <0.0001* |
|  | XIX Injury, poisoning and certain other consequences of external causes | 125/826 | 1.67 (1.25-2.24) | 0.001* |
| From mild to moderate AD dementia | XVIII Symptoms, signs and abnormal clinical and laboratory findings, not elsewhere classified | 1212/3181 | 0.90 (0.83-0.99) | 0.037* |
| From mild AD dementia to institutionalization | V Mental, Behavioral and Neurodevelopmental disorders | 158/751 | 1.42 (1.18-1.70) | 0.000* |
|  | XIX Injury, poisoning and certain other consequences of external causes | 383/1799 | 1.39 (1.18-1.62) | <0.0001* |
| From mild AD dementia to death | IV Endocrine, nutritional and metabolic diseases | 80/1004 | 1.45 (1.05-2.01) | 0.024* |
|  | V Mental, Behavioral and Neurodevelopmental disorders | 56/649 | 2.23 (1.60-3.08) | <0.0001* |
|  | IX Diseases of the circulatory system | 126/1729 | 1.55 (1.06-2.26) | 0.023* |
| From moderate to severe AD dementia | XIX Injury, poisoning and certain other consequences of external causes | 370/1639 | 0.88 (0.76-1.00) | 0.056 |
|  | XXI Factors influencing health status and contact with health services | 698/2883 | 0.83 (0.69-0.99) | 0.036* |
| From moderate AD dementia to institutionalization | I Certain infectious and parasitic diseases | 302/755 | 1.19 (1.04-1.35) | 0.010* |
|  | V Mental, Behavioral and Neurodevelopmental disorders | 357/905 | 1.30 (1.15-1.47) | <0.0001* |
| From moderate AD dementia to death | II Neoplasms | 102/1026 | 1.67 (1.25-2.21) | 0.001* |
|  | III Diseases of the blood and blood-forming organs and certain disorders involving the immune mechanism | 38/266 | 1.72 (1.18-2.48) | 0.004* |
|  | IX Diseases of the circulatory system | 141/1518 | 1.52 (1.09-2.12) | 0.014* |
| From severe AD dementia to institutionalization | VIII Diseases of the ear and mastoid process | 147/218 | 0.79 (0.66-0.96) | 0.016* |
| From institutionalized very mild AD dementia to institutionalized mild AD dementia | II Neoplasms | 12/92 | 0.53 (0.25-1.11) | 0.091 |
| From institutionalized very mild AD dementia to death | III Diseases of the blood and blood-forming organs and certain disorders involving the immune mechanism | 8/33 | 4.53 (1.58-12.97) | 0.005* |
| From institutionalized mild AD dementia to institutionalized moderate AD dementia | IX Diseases of the circulatory system | 60/486 | 0.63 (0.40-0.98) | 0.041* |
| From institutionalized mild AD dementia to death | II Neoplasms | 37/266 | 1.70 (1.03-2.77) | 0.037* |
|  | X Diseases of the respiratory system | 31/184 | 2.20 (1.35-3.61) | 0.002* |
| From institutionalized moderate AD dementia to institutionalized severe AD dementia | VIII Diseases of the ear and mastoid process | 45/315 | 0.71 (0.51-0.98) | 0.039* |
| From institutionalized moderate AD dementia to death | II Neoplasms | 70/491 | 1.46 (1.06-2.03) | 0.022* |
|  | VI Diseases of the nervous system | 61/396 | 1.43 (1.03-1.99) | 0.035* |
| From institutionalized severe AD dementia to death | IX Diseases of the circulatory system | 423/618 | 1.28 (1.09-1.51) | 0.003* |

Abbreviations: AD = Alzheimer’s disease; CI = confidence interval; CSF = cerebrospinal fluid; HR = hazard ratio.

Notes: ICD codes related to dementia (F00, F01, F02, F03, F05, F06, G30, G31, and R41) were excluded from chapters V. The results were from the multistate model including all the comorbidities in the table as covariates.

The results were derived from multistate models adjusted for age, sex, Aβ_42_, P-tau_181_, and T-tau, incorporating all comorbidities associated with AD dementia stages as listed in Table S5.

*P-value <0.05.

# Table S8. Interactions between selected comorbidities and age and sex

| Transition | Predictor | HR (95% CI) for predictor | HR (95% CI) for interaction with age | HR (95% CI) for interaction with sex |
| --- | --- | --- | --- | --- |
| From very mild to mild AD dementia | T2DM | 0.79 (0.59-1.06) | 1.00 (0.97-1.03) | 0.95 (0.62-1.46) |
|  | IHD | 0.90 (0.71-1.16) | 0.99 (0.97-1.02) | 1.31 (0.91-1.87) |
|  | Stroke | 0.76 (0.52-1.11) | 0.99 (0.95-1.04) | 1.23 (0.74-2.05) |
|  | Depression | 0.98 (0.73-1.31) | 1.03 (1.00-1.05)* | 1.20 (0.83-1.72) |
| From very mild AD dementia to institutionalization | T2DM | 1.54 (0.88-2.67) | 0.95 (0.90-1.01) | 0.58 (0.24-1.42) |
|  | IHD | 0.80 (0.42-1.54) | 1.03 (0.97-1.10) | 1.45 (0.66-3.18) |
|  | Stroke | - | - | - |
|  | Depression | 2.66 (1.54-4.59) | 0.98 (0.93-1.03) | 0.62 (0.30-1.28) |
| From mild to moderate AD dementia | T2DM | 0.90 (0.72-1.14) | 1.01 (0.98-1.03) | 1.11 (0.79-1.54) |
|  | IHD | 0.86 (0.71-1.04) | 1.02 (1.00-1.04)* | 1.08 (0.82-1.45) |
|  | Stroke | 0.96 (0.72-1.28) | 1.03 (0.99-1.06) | 0.79 (0.52-1.22) |
|  | Depression | 1.01 (0.80-1.27) | 1.02 (1.00-1.03) | 0.90 (0.68-1.22) |
| From mild AD dementia to institutionalization | T2DM | 1.67 (1.21-2.30) | 1.00 (0.97-1.04) | 0.77 (0.50-1.21) |
|  | IHD | 0.73 (0.52-1.02) | 1.02 (0.99-1.06) | 1.73 (1.15-2.63)* |
|  | Stroke | 1.63 (1.12-2.38) | 1.00 (0.96-1.05) | 0.84 (0.51-1.39) |
|  | Depression | 1.32 (0.89-1.98) | 1.01 (0.97-1.04) | 0.75 (0.45-1.22) |
| From mild AD dementia to death | T2DM | 1.45 (0.80-2.61) | 1.02 (0.95-1.10) | 0.82 (0.33-2.04) |
|  | IHD | 0.88 (0.50-1.53) | 1.03 (0.98-1.10) | 2.69 (1.32-5.47)* |
|  | Stroke | 2.12 (1.15-3.88) | 0.97 (0.90-1.05) | 0.82 (0.31-2.15) |
|  | Depression | 2.08 (1.16-3.69) | 0.97 (0.92-1.03) | 1.21 (0.54-2.70) |
| From moderate to severe AD dementia | T2DM | 0.90 (0.63-1.29) | 1.00 (0.97-1.04) | 1.04 (0.61-1.76) |
|  | IHD | 1.00 (0.75-1.33) | 0.99 (0.96-1.02) | 0.85 (0.53-1.36) |
|  | Stroke | 0.84 (0.50-1.40) | 1.05 (1.00-1.12)** | 0.87 (0.44-1.72) |
|  | Depression | 0.92 (0.65-1.31) | 1.00 (0.98-1.03) | 0.85 (0.54-1.33) |
| From moderate AD dementia to institutionalization | T2DM | 1.09 (0.86-1.39) | 1.02 (0.99-1.04) | 1.39 (1.01-1.92)* |
|  | IHD | 0.92 (0.74-1.15) | 0.99 (0.97-1.02) | 1.22 (0.91-1.64) |
|  | Stroke | 1.12 (0.82-1.52) | 1.01 (0.98-1.04) | 0.98 (0.66-1.45) |
|  | Depression | 1.25 (0.96-1.60) | 0.99 (0.97-1.02) | 0.99 (0.72-1.36) |
| From moderate AD dementia to death | T2DM | 1.19 (0.62-2.26) | 1.02 (0.95-1.09) | 0.90 (0.35-2.30) |
|  | IHD | 1.08 (0.61-1.91) | 0.99 (0.93-1.05) | 1.30 (0.61-2.74) |
|  | Stroke | - | - | - |
|  | Depression | 1.36 (0.70-2.66) | 0.99 (0.92-1.06) | 0.77 (0.31-1.91) |
| From severe AD dementia to institutionalization | T2DM | 0.84 (0.56-1.27) | 1.04 (1.01-1.08)* | 1.34 (0.78-2.28) |
|  | IHD | 0.99 (0.69-1.40) | 0.98 (0.95-1.02) | 0.79 (0.47-1.34) |
|  | Stroke | 0.70 (0.42-1.20) | 1.01 (0.96-1.07) | 1.70 (0.90-3.19) |
|  | Depression | 0.91 (0.65-1.27) | 0.96 (0.94-0.99)* | 0.86 (0.55-1.33) |
| From severe AD dementia to death | T2DM | - | - | - |
|  | IHD | 2.08 (1.14-3.79) | 0.93 (0.88-0.99)* | 0.40 (0.11-1.48) |
|  | Stroke | - | - | - |
|  | Depression | 0.85 (0.39-1.84) | 0.94 (0.89-1.01)** | 1.17 (0.44-3.16) |
| From institutionalized mild AD dementia to death | T2DM | - | - | - |
|  | IHD | 1.62 (0.77-3.39) | 1.07 (0.99-1.16)** | 1.97 (0.67-5.76) |
|  | Stroke | - | - | - |
|  | Depression | - | - | - |
| From institutionalized moderate AD dementia to institutionalized severe AD dementia | T2DM | 0.88 (0.48-1.62) | 1.04 (0.98-1.11) | 1.00 (0.45-2.20) |
|  | IHD | 1.15 (0.71-1.84) | 1.00 (0.95-1.05) | 0.94 (0.47-1.86) |
|  | Stroke | 0.94 (0.47-1.86) | 0.99 (0.92-1.06) | 0.97 (0.39-2.39) |
|  | Depression | 0.86 (0.47-1.57) | 0.99 (0.95-1.04) | 0.97 (0.46-2.02) |
| From institutionalized moderate AD dementia to death | T2DM | 1.32 (0.77-2.28) | 0.96 (0.90-1.02) | 0.75 (0.31-1.81) |
|  | IHD | 1.30 (0.76-2.21) | 0.95 (0.89-1.01)** | 1.26 (0.55-2.84) |
|  | Stroke | - | - | - |
|  | Depression | 1.21 (0.67-2.18) | 0.96 (0.91-1.03) | 0.99 (0.42-2.32) |
| From institutionalized severe AD dementia to death | T2DM | 1.62 (1.14-2.30) | 0.99 (0.96-1.02) | 0.72 (0.45-1.16) |
|  | IHD | 0.99 (0.71-1.37) | 1.00 (0.97-1.03) | 1.36 (0.87-2.13) |
|  | Stroke | 0.97 (0.60-1.57) | 0.99 (0.94-1.03) | 0.94 (0.53-1.66) |
|  | Depression | 0.90 (0.65-1.28) | 0.99 (0.96-1.02) | 1.02 (0.65-1.60) |

Abbreviations: AD = Alzheimer’s disease; CI = confidence interval; HR = hazard ratio; IHD = ischemic heart disease; T2DM = type 2 diabetes.

Notes: The results were derived from multistate models that included all comorbidities listed in the table as covariates.

Transitions with <20 events were excluded from the analyses, including transitions from very mild AD dementia to death, from institutionalized very mild AD dementia to mild AD dementia, from institutionalized very mild AD dementia to death, and from institutionalized mild AD dementia to moderate AD dementia.

*P-value for interaction <0.05.

**P-value for interaction <0.1.

# Table S9. The associations between selected comorbidities and AD dementia prognosis, adjusted for CSF biomarkers

| Transition | Predictor | No. events/No. people at risk | HR (95% CI) | P-value |
| --- | --- | --- | --- | --- |
| From very mild to mild AD dementia | T2DM | 98/241 | 0.79 (0.64-0.97) | 0.027* |
|  | IHD | 140/318 | 1.01 (0.84-1.21) | 0.915 |
|  | CKD | 9/29 | 0.84 (0.44-1.63) | 0.616 |
|  | Stroke | 64/160 | 0.81 (0.63-1.04) | 0.101 |
|  | Depression | 143/287 | 1.05 (0.88-1.25) | 0.598 |
|  | IBD | 13/26 | 1.42 (0.82-2.46) | 0.207 |
| From very mild AD dementia to institutionalization | T2DM | 25/168 | 1.15 (0.75-1.77) | 0.519 |
|  | IHD | 30/208 | 1.07 (0.72-1.61) | 0.715 |
|  | CKD | 3/23 | 1.04 (0.33-3.31) | 0.942 |
|  | Stroke | 17/113 | 1.01 (0.61-1.68) | 0.968 |
|  | Depression | 37/181 | 1.99 (1.39-2.86) | 0.000* |
|  | IBD | 3/16 | 1.65 (0.53-5.19) | 0.389 |
| From very mild AD dementia to death | T2DM | 5/148 | 0.73 (0.29-1.86) | 0.512 |
|  | IHD | 14/192 | 1.82 (0.96-3.44) | 0.068 |
|  | CKD | 3/23 | 2.86 (0.85-9.61) | 0.091 |
|  | Stroke | 5/101 | 1.05 (0.41-2.70) | 0.924 |
|  | Depression | 6/150 | 1.25 (0.52-2.99) | 0.626 |
|  | IBD | 2/15 | 3.25 (0.77-13.83) | 0.109 |
| From mild to moderate AD dementia | T2DM | 153/426 | 0.96 (0.81-1.13) | 0.608 |
|  | IHD | 231/643 | 0.92 (0.80-1.07) | 0.281 |
|  | CKD | 14/51 | 0.72 (0.43-1.23) | 0.229 |
|  | Stroke | 94/272 | 0.93 (0.76-1.15) | 0.535 |
|  | Depression | 216/538 | 0.92 (0.80-1.07) | 0.281 |
|  | IBD | 34/74 | 1.06 (0.75-1.49) | 0.741 |
| From mild AD dementia to institutionalization | T2DM | 95/368 | 1.42 (1.14-1.77) | 0.002* |
|  | IHD | 113/525 | 1.07 (0.87-1.32) | 0.504 |
|  | CKD | 14/51 | 1.43 (0.84-2.44) | 0.185 |
|  | Stroke | 72/250 | 1.54 (1.20-1.98) | 0.001* |
|  | Depression | 76/398 | 1.13 (0.88-1.43) | 0.346 |
|  | IBD | 10/50 | 1.15 (0.62-2.15) | 0.657 |
| From mild AD dementia to death | T2DM | 26/299 | 1.42 (0.92-2.17) | 0.113 |
|  | IHD | 44/456 | 1.48 (1.04-2.11) | 0.029* |
|  | CKD | 10/47 | 3.25 (1.70-6.22) | 0.000* |
|  | Stroke | 23/201 | 1.80 (1.15-2.82) | 0.010* |
|  | Depression | 30/352 | 1.93 (1.31-2.89) | 0.001* |
|  | IBD | 1/41 | 0.40 (0.06-2.90) | 0.367 |
| From moderate to severe AD dementia | T2DM | 62/297 | 0.86 (0.66-1.12) | 0.267 |
|  | IHD | 88/421 | 0.95 (0.76-1.19) | 0.673 |
|  | CKD | 7/36 | 0.87 (0.41-1.83) | 0.708 |
|  | Stroke | 36/200 | 0.85 (0.61-1.20) | 0.368 |
|  | Depression | 93/375 | 0.85 (0.69-1.06) | 0.156 |
|  | IBD | 10/48 | 0.76 (0.40-1.41) | 0.375 |
| From moderate AD dementia to institutionalization | T2DM | 177/412 | 1.34 (1.14-1.58) | 0.000* |
|  | IHD | 216/549 | 1.01 (0.87-1.17) | 0.900 |
|  | CKD | 12/41 | 0.89 (0.50-1.57) | 0.684 |
|  | Stroke | 112/276 | 1.09 (0.90-1.34) | 0.344 |
|  | Depression | 194/476 | 1.26 (1.08-1.47) | 0.003* |
|  | IBD | 23/61 | 0.98 (0.65-1.49) | 0.939 |
| From moderate AD dementia to death | T2DM | 26/261 | 1.22 (0.80-1.88) | 0.355 |
|  | IHD | 41/374 | 1.11 (0.77-1.59) | 0.570 |
|  | CKD | 8/37 | 2.20 (1.07-4.57) | 0.032* |
|  | Stroke | 18/182 | 1.15 (0.70-1.88) | 0.584 |
|  | Depression | 22/304 | 1.21 (0.77-1.90) | 0.417 |
|  | IBD | 3/41 | 1.17 (0.38-3.70) | 0.778 |
| From severe AD dementia to institutionalization | T2DM | 67/91 | 1.00 (0.77-1.30) | 0.994 |
|  | IHD | 75/103 | 0.90 (0.70-1.15) | 0.388 |
|  | CKD | 10/16 | 1.46 (0.78-2.76) | 0.233 |
|  | Stroke | 43/53 | 0.97 (0.71-1.33) | 0.842 |
|  | Depression | 95/139 | 0.86 (0.69-1.07) | 0.174 |
|  | IBD | 8/14 | 0.76 (0.37-1.52) | 0.431 |
| From severe AD dementia to death | T2DM | 18/42 | 1.30 (0.76-2.20) | 0.347 |
|  | IHD | 23/51 | 1.27 (0.79-2.06) | 0.315 |
|  | CKD | 1/7 | 0.61 (0.08-4.39) | 0.621 |
|  | Stroke | 14/24 | 1.27 (0.72-2.22) | 0.410 |
|  | Depression | 20/64 | 0.73 (0.45-1.18) | 0.203 |
|  | IBD | 4/10 | 1.11 (0.39-3.14) | 0.852 |
| From institutionalized very mild AD dementia to institutionalized mild AD dementia | T2DM | 4/31 | 1.21 (0.39-3.73) | 0.736 |
|  | IHD | 11/35 | 2.23 (1.04-4.77) | 0.039* |
|  | CKD | 0/3 | - | 0.977 |
|  | Stroke | 1/28 | 0.18 (0.02-1.37) | 0.097 |
|  | Depression | 10/48 | 1.32 (0.61-2.85) | 0.475 |
|  | IBD | 0/3 | - | 0.977 |
| From institutionalized very mild AD dementia to death | T2DM | 3/30 | 1.15 (0.31-4.30) | 0.836 |
|  | IHD | 3/27 | 0.98 (0.26-3.74) | 0.974 |
|  | CKD | 1/4 | 2.36 (0.30-18.87) | 0.415 |
|  | Stroke | 3/30 | 0.95 (0.27-3.42) | 0.944 |
|  | Depression | 3/41 | 1.05 (0.29-3.85) | 0.937 |
|  | IBD | 0/3 | - | 0.987 |
| From institutionalized mild AD dementia to institutionalized moderate AD dementia | T2DM | 14/107 | 1.13 (0.63-2.01) | 0.698 |
|  | IHD | 13/120 | 1.00 (0.54-1.82) | 0.987 |
|  | CKD | 1/18 | 0.73 (0.10-5.45) | 0.765 |
|  | Stroke | 8/87 | 0.71 (0.34-1.49) | 0.369 |
|  | Depression | 19/110 | 0.97 (0.57-1.64) | 0.899 |
|  | IBD | 1/12 | 0.52 (0.07-3.72) | 0.510 |
| From institutionalized mild AD dementia to death | T2DM | 11/104 | 0.83 (0.41-1.65) | 0.587 |
|  | IHD | 27/134 | 2.39 (1.42-4.01) | 0.001* |
|  | CKD | 3/20 | 1.73 (0.53-5.71) | 0.364 |
|  | Stroke | 13/92 | 1.07 (0.56-2.06) | 0.834 |
|  | Depression | 14/105 | 1.27 (0.69-2.34) | 0.438 |
|  | IBD | 1/12 | 0.57 (0.08-4.13) | 0.573 |
| From institutionalized moderate AD dementia to institutionalized severe AD dementia | T2DM | 31/196 | 0.91 (0.62-1.34) | 0.636 |
|  | IHD | 42/226 | 1.15 (0.82-1.61) | 0.427 |
|  | CKD | 4/16 | 1.75 (0.64-4.82) | 0.272 |
|  | Stroke | 23/147 | 0.95 (0.61-1.47) | 0.808 |
|  | Depression | 39/230 | 0.84 (0.60-1.19) | 0.340 |
|  | IBD | 3/28 | 0.93 (0.30-2.92) | 0.903 |
| From institutionalized moderate AD dementia to death | T2DM | 29/194 | 1.11 (0.72-1.71) | 0.634 |
|  | IHD | 32/216 | 1.09 (0.72-1.65) | 0.673 |
|  | CKD | 7/19 | 3.39 (1.48-7.80) | 0.004* |
|  | Stroke | 19/143 | 0.93 (0.56-1.54) | 0.775 |
|  | Depression | 27/218 | 1.17 (0.77-1.80) | 0.450 |
|  | IBD | 1/26 | 0.64 (0.09-4.62) | 0.660 |
| From institutionalized severe AD dementia to death | T2DM | 85/117 | 1.31 (1.04-1.67) | 0.023* |
|  | IHD | 100/134 | 1.14 (0.92-1.43) | 0.237 |
|  | CKD | 12/17 | 1.63 (0.90-2.92) | 0.104 |
|  | Stroke | 57/88 | 0.88 (0.67-1.16) | 0.368 |
|  | Depression | 89/152 | 0.92 (0.74-1.15) | 0.484 |
|  | IBD | 7/12 | 1.32 (0.62-2.81) | 0.470 |

Abbreviations: AD = Alzheimer’s disease; CI = confidence interval; CKD = chronic kidney disease; CSF = cerebrospinal fluid; HR = hazard ratio; IBD = inflammatory bowel disease; IHD = ischemic heart disease; T2DM = type 2 diabetes.

The results were derived from multistate models adjusted for age, sex, Aβ_42_, P-tau_181_, and T-tau, incorporating all comorbidities associated with AD dementia stages as listed in the table.

P-value<0.05.
